# Educational materials to empower parents of preterm infants within a family-centered early intervention in the NICU

**DOI:** 10.3389/fped.2026.1823643

**Published:** 2026-06-09

**Authors:** Chiara Bonfanti, Camilla Fontana, Sara Meloni, Fabio Mosca, Monica Fumagalli

**Affiliations:** 1 Fondazione IRCCS Ca' Granda Ospedale Maggiore Policlinico, NICU, Milan, Italy; 2 Department of Clinical Sciences and Community Health, Dipartimento di Eccellenza 2023-2027, University of Milan, Milan, Italy

**Keywords:** early intervention, educational materials, family-centered care, neurodevelopment, NICU, parent education, prematurity

## Abstract

**Introduction:**

Prematurity exposes infants and families to multiple stressors during NICU stay, with potential effects on neurodevelopment and parental wellbeing. Evidence highlights the protective role of early interventions (EI) that actively involve parents. This project aimed to design and introduce tailored educational materials for parents of preterm infants to support a structured EI program during NICU stay.

**Methods:**

The project was part of a clinical study including biparental families of preterm infants born between 24^+0^ and 32^+6^ weeks of gestation, who received a previously designed EI program based on parental involvement and multisensory experiences. Following contextual analysis and existing literature, educational materials were developed through iterative focus groups involving NICU professionals and incorporating parents' informal feedback. After the initial definition of content and design, multiple drafts were reviewed to optimize clarity, accessibility, and layout.

**Results:**

Seven bilingual (Italian/English) leaflets were developed, addressing infant behavioral cues, co-regulation, positive multisensory experiences (voice listening, massage therapy, visual interaction), and transition at home. The materials were shared with 104 families (mean GA = 29.95 ± 2.11 weeks). Parents highlighted the strong emotional resonance of the NICU experience and the perceived value of the materials. Professionals informally reported improved coherence in EI delivery and perceived enhanced collaboration with NICU staff.

**Discussion:**

Educational materials can strengthen parental engagement when implementing EI programs in the NICU, which is of paramount importance to create a nurturing environment. Future research is needed to validate the effectiveness of these resources and assess their impact on parent-infant outcomes.

## 1 Introduction

Prematurity, accounting for 9.9% of all livebirths (1), is a complex condition that affects both the preterm infant and the entire family system, as they go through a delicate period in the Neonatal Intensive Care Unit (NICU), facing multiple clinical and environmental stressors (2).

In the NICU, preterm infants are frequently exposed to early adverse experiences, including pain, excessive sensory stimulation (i.e., loud noise, intense light) (3, 4), and deprivation of the physiological intrauterine sensory environment, often combined with prolonged parental separation and reduced parental care (5). These stressors occur during a critical period of heightened neuroplasticity, when brain development is highly sensitive to environmental influences (6, 7). As a result, early life adversity in the NICU can interfere with the sensory-driven brain maturation and stress-regulatory systems (8–10), contributing to the well-known brain alterations (11) and increased risk for later neurodevelopmental delays observed in prematurely born children (2, 12, 13).

At the same time, parents of preterm infants commonly encounter significant emotional challenges in developing their parental role, including symptoms of depression, anxiety, and post-traumatic stress, which can persist beyond hospital discharge (14). Moreover, the unexpected medical complexity of the preterm infant may disrupt parents' expectations of childbirth and early parenthood, negatively affecting their sensitivity, responsiveness, and attachment (15). Such parental distress, together with contextual barriers to early bonding, can negatively affect parent–infant interactions (6) and has been linked to both behavioral outcomes (16) and brain development (17) in preterm children.

Overall, the NICU environment and associated stressors are thought to exert a profound impact on the entire family, with repercussions on infant neurodevelopment (2) and parental psychological well-being (18).

To counteract these detrimental effects, increasing attention has been directed toward parent-focused early interventions (EI) (19). Early sensitive caregiving has been shown to positively influence brain maturation in preterm-born children, with a protective effect on neurodevelopment even after accounting for medical risk factors such as brain injury (17, 20). Accordingly, recent evidence underscores the central role of the family and the importance of supporting the early parent-infant relationship in mitigating prematurity-related stress (19).

To foster attuned and responsive parent-child interactions and dyadic co-regulation, EI strategies in the NICU should prioritize parental involvement within a family-centred care framework (21–23). Understanding the needs and perspectives of parents themselves is essential to promote such engagement effectively. Research studies indicate that parents' informational needs evolve over NICU stay and may be influenced by infant gestational age, the length of the hospitalization, parental age and previous parenting experience (24–26). Core content areas of parental needs include understanding the infant's medical course and care, accessing psychosocial support, learning how to read and act on infants' cues, and strategies for coping with the NICU experience (24, 25, 27).

Educational initiatives based on a multilayered approach are recommended as a way to meet the complex and evolving needs of parents (28–30). Given the overwhelming amount of medical information that families receive during the NICU stay, offering content through diverse methods (such as written handouts, videos, and interactive observation and discussion) can enhance understanding and accommodate varying learning preferences (28, 31). Therefore, the introduction of tailored written resources could serve as a valuable complement to verbal discussions, improving consistency and strengthening a continuous dialogue between families and healthcare professionals (32). Programs such as SENSE (33, 34) and COPE (35) have effectively integrated multimodal educational tools to actively engage parents in developmentally supportive caregiving. Nonetheless, only few studies have explored the developing process of parent-education tools, particularly in the NICU context (36–39). Although educational resources designed to support parents in other healthcare settings exist (40–42), they may not adequately address the specific emotional and informational needs of NICU parents.

The present project is part of a larger study investigating the relationship between EI based on parental involvement and multisensory experiences, long-term neurobehavioral outcomes and epigenetic modifications. The current work describes the development of educational resources to support the EI program and encourage parents' active involvement in the care of their newborns during NICU stay.

## 2 Material and methods

### 2.1 Study design

This project is part of a clinical, non-pharmacological, biological study, entitled “Structural variations of the neural genome as prognostic biomarkers for prematurity related neurodevelopmental disorders in childhood” performed at Fondazione IRCCS Ca' Granda Ospedale Maggiore Policlinico of Milan. Within the study, all the enrolled preterm infants born between 24^+0^ and 32^+6^ weeks of gestational age received a structured EI program during NICU stay, based on early parental involvement and multisensory interactions, conceived and evaluated in a preliminary randomized controlled trial (43) and adapted from the PremieStart Protocol (44). The intervention was addressed to biparental families. The EI program was delivered by specifically trained neurodevelopmental therapists and consisted in three parts related to each other: parental training, massage therapy, and visual interaction [for further details see (43)]. The enriched multisensory experiences were performed by parents after a training period. The educational materials were designed as active tools to support the parental training sessions and promote parental empowerment.

The protocol has been approved by the Ethical Committee Milano Area B on the 4th of June 2019 (approval number 530_2019). The trial is registered at clinicaltrials.gov (Trial Registration Number: NCT04617587).

### 2.2 Procedure

**Figure 1:**
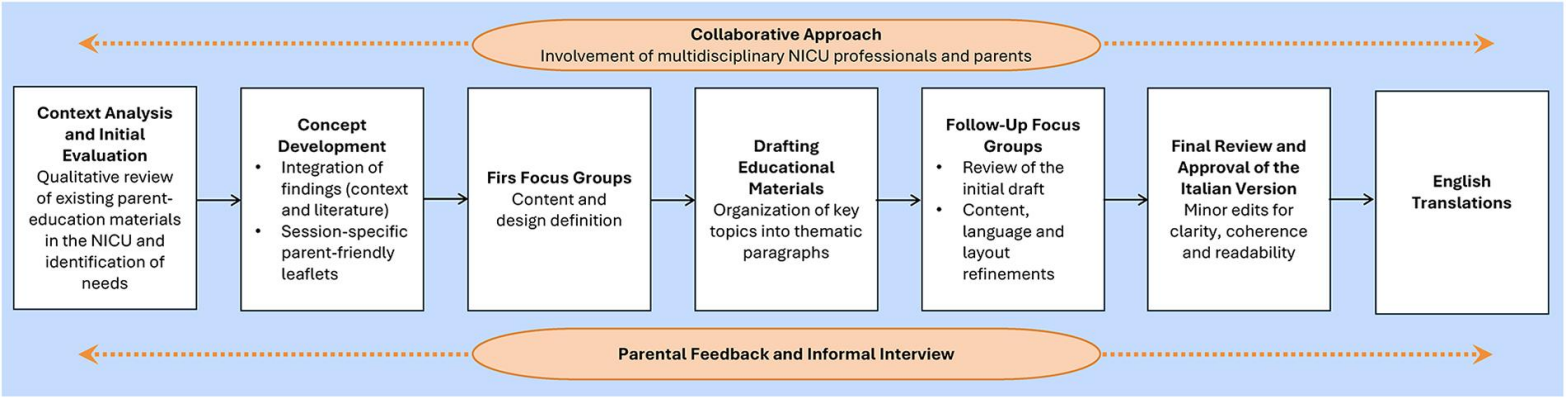
Overview of the key phases in the development of the educational materials.

The development of educational materials to support the EI program followed a structured iterative process (38, 40) that unfolded through key phases: an initial contextual analysis and identification of needs, iterative drafting and revision rounds informed by multidisciplinary focus groups and ongoing informal parental feedback, and the final introduction of the materials within the EI program, followed by unstructured feedback collection from both professionals and families involved. All these phases are outlined in the section below and summarized in Figure 1.

First, a critical review of the parent-education materials currently used in the unit was conducted by the multidisciplinary team. In the context of the Level III NICU at Ospedale Maggiore Policlinico of Milan, educational resources were provided to parents of preterm infants in the form of a booklet containing information on preterm infants' sensory and motor development, as well as strategies for developmental care and positioning, covering the period from birth through discharge (45).

Within the framework of the previously conceived EI intervention (43), the need to enhance the EI program with a more structured, but still adaptable, educational approach became evident.

Based on this contextual analysis and existing literature, the introduction of tailored written educational materials, addressing both infant care and parents' informational needs, emerged as the most appropriate strategy. To support a parent-friendly communication, the chosen modality consisted of session-specific leaflets, designed to gradually introduce information in line with the EI program progression. The materials were introduced as soon as families provided informed consent to participate in the study and handed out one-by-one during the training sessions in the NICU. After each session, the leaflets were left with the families, allowing them to revisit the content and reinforce key concepts at their own pace.

The second phase of the project consisted of successive focus groups involving different NICU professionals to define content and design of the educational materials. A collaborative approach was adopted, incorporating inputs from an academic researcher from the University of Milan in the field of early intervention and neurorehabilitation and senior neurodevelopmental therapists with expertise in early intervention for preterms (who were directly involved in the study research team and in the delivery of the EI program), and nurses and neonatologists of the NICU team. All the healthcare professionals involved had clinical experience in the NICU and were familiar with clinical research. Additionally, iterative feedback from families involved in the previous EI experience (43), as well as from those participating in the present study, was considered. The focus groups discussions were guided by the principles of the Family-Centered Care (21).

As a first step, a structured outline was prepared, listing possible titles and key topics for each leaflet. In line with our EI program method (43) (based on PremieStart Protocol), an initial set of nine leaflets was hypothesized, covering the following themes: stress and availability signals, behavioral states, self-regulation behaviors, postural care, gentle handling and containment, voice listening, infant massage, visual interaction, and interactions after discharge. Following discussion, it was agreed to reduce the number of leaflets to avoid overwhelming parents with an excessive number of materials. We therefore decided to integrate the content on self-regulation behaviors and gentle handling and containment within the leaflets on stress/availability signals and behavioral states, rather than developing separate brochures. Consideration was also given to the stratification of proposals by gestational age, as suggested in comparable materials (33): however, a non-stratified approach was preferred, to further promote individualized care, allowing parents in modulating interactions and caregiving proposals based on their infant's unique signals and their own preferences and needs. Gestational age indications were nonetheless included in the massage and visual interaction leaflets in the form of minimum age thresholds (e.g., “not before … weeks postmenstrual age”), reflecting the specific protocol requirements for infant massage and the maturational constraints on visual function.

The information gathered through these discussions, in combination with existing literature and the prior EI program, guided the development of an initial written draft of the leaflets. This draft outlined the parental training sessions, with content organized into thematic sections addressing different key aspects of infant behavior and positive multisensory interactions (voice, massage, and visual).

Following completion of the initial draft, follow-up focus groups were held to review the content and incorporate recommended amendments. Discussions and consultations focused on evaluating the content's suitability, accuracy, and overall structure. Particular attention was paid to the language used, the clarity and conciseness of the information, and accessibility across different parental education levels, including through the incorporation of images (38).

Iterative content revisions were informed by feedback from the multidisciplinary team as well as from the parents. Throughout the whole process, informal semi-structured interviews with parents were conducted to tailor the materials to their individual feedback and needs. During the educational sessions, they were encouraged to share their opinions and suggestions though predefined themes and questions, including whether they had reviewed the leaflet from the previous session, whether the content was clear and useful for understanding their infant's signals and supporting interaction, and whether they had any suggestions regarding format and usability, while allowing for flexibility based on each family's availability and engagement with the intervention. Their feedback was used to iteratively adjust the content and the layout of the leaflets.

Specific additions and refinements included: the addition of some items to the stress/availability signals leaflets; adjustments to the language used to describe co-regulation strategies, emphasizing environmental modulation in response to infant cues; clarification of optimal timing and conditions for each proposed interaction (e.g., behavioral state, distance from feeding, environmental quietness); the integration of postural support recommendations within the multisensory interaction leaflets; the inclusion of safe sleep guidance and prone positioning recommendations within the posture leaflet; and refinements to the discharge leaflet to reflect the transition to home, including guidance on play, varied holding positions, and environmental conditions conducive to interaction.

The section headings for the multisensory interaction leaflets were harmonized to consistently frame each modality as a means of promoting neurodevelopment through multisensory experience, rather than targeting a single sensory domain. Additional revisions were made to refine the graphic design and presentation of the materials, while retaining the core messages and ensuring clarity and ease of use.

In the final stages, attention was given to the visual aspects of the leaflets. Initially, generic photos were used from accessible materials already available in the hospital (45). The involvement of families in the study allowed for the inclusion of photographs of the actual participants, increasing their sense of empowerment and participation in the program. This ensured the use of ecologically valid images, enhancing the credibility and relevance of the leaflets for the target population. Written consent by both parents was obtained for the use of infants' pictures and their parents. An acknowledgment of the families and infants was included at the end of each leaflet.

In the last focus group, minor edits were implemented to enhance coherence and readability, and the final draft was approved.

The materials were originally developed in Italian; to ensure accessibility for a wider audience, an English translation was subsequently performed.

Following the introduction of the leaflets within the EI program, informal feedback was additionally gathered from both parents and neurodevelopmental therapists involved in the project. Parental feedback was collected during the post-discharge follow-up visit included in the EI program. Therapists' feedback was gathered during final focus groups conducted during and after the introduction of the educational resources.

## 3 Results

The final materials consisted of seven French-fold brochures, available in both Italian and English versions (see the Supplementary Material), developed to support one-to-one EI sessions.

Each leaflet was dedicated to a specific topic within the parental training program, allowing for a step-by-step shared pathway during NICU stay.

**Figure 2:**
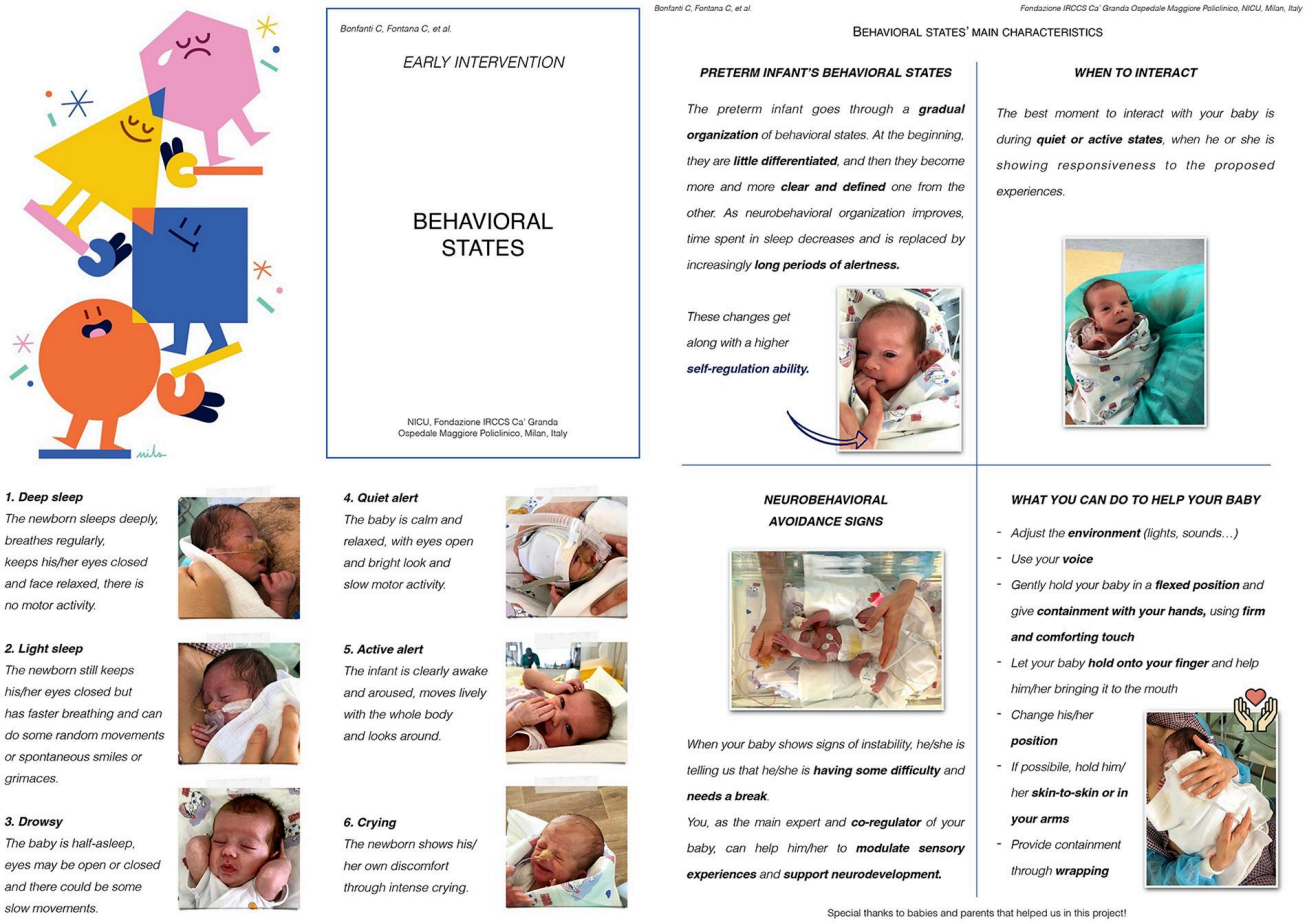
Example of one leaflet focusing on infant's behavioral states.

Specifically: three leaflets focused on infant behavior (identification of newborn's signs of stress or availability and behavioral states), facilitation strategies to respond to infant's cues, and postural care; three leaflets focused on co-regulation, behavioral modulation and multisensory interactions (voice listening, infant's massage, and visual interaction); one leaflet provided guidance in preparation for transition to home. An illustrative example of one leaflet is shown in Figure 2.

The final version of the leaflets shared a consistent overall layout, making the sequence of information clear and predictable for the readers. The contents were organized into headers and concise sections, including a brief description of the topic, indications on when and how to perform enriched interactions, and additional tips or precautions, with key information highlighted in bold.

Short sentences and simple language were preferred, avoiding difficult or uncommon words and using an action-oriented lexicon with clear instructions for incorporating activities into daily care. Particular attention was paid to the wording used to describe parent-infant interactions, to minimize parental stress or anxiety. Terms were carefully selected to acknowledge that not all activities may be suitable for every infant, with suggested adaptations based on individual characteristics.

In the Italian version, the second-person pronoun “you” was chosen to create a personal and confident tone, better mirroring the everyday speech and helping the caregiver to feel at ease, thereby complementing the in-person educational sessions.

Graphics and visuals were incorporated matching the text and the different leaflets' sections, to clarify the concepts presented and to encourage the active involvement of the families. Actual photographs, along with bright colors and engaging images, were accurately selected to convey a sense of authenticity and to help parents relate the brochure content to real-life experiences, also improving the overall appeal of the materials.

The set of leaflets was also made available in English. The translation process closely mirrored the development of the original Italian version, maintaining consistency in tone, structure, and content. Once the text was translated, it was integrated into the existing graphic layout, with adjustments made as needed to preserve the visual coherence and communicative effectiveness of the materials. The aim of providing an English version was twofold: to broaden the accessibility of the materials to a wider range of families, and to facilitate dissemination and research collaboration at the international level, allowing the use of the leaflets as supportive tools in diverse contexts.

**Figure 3:**
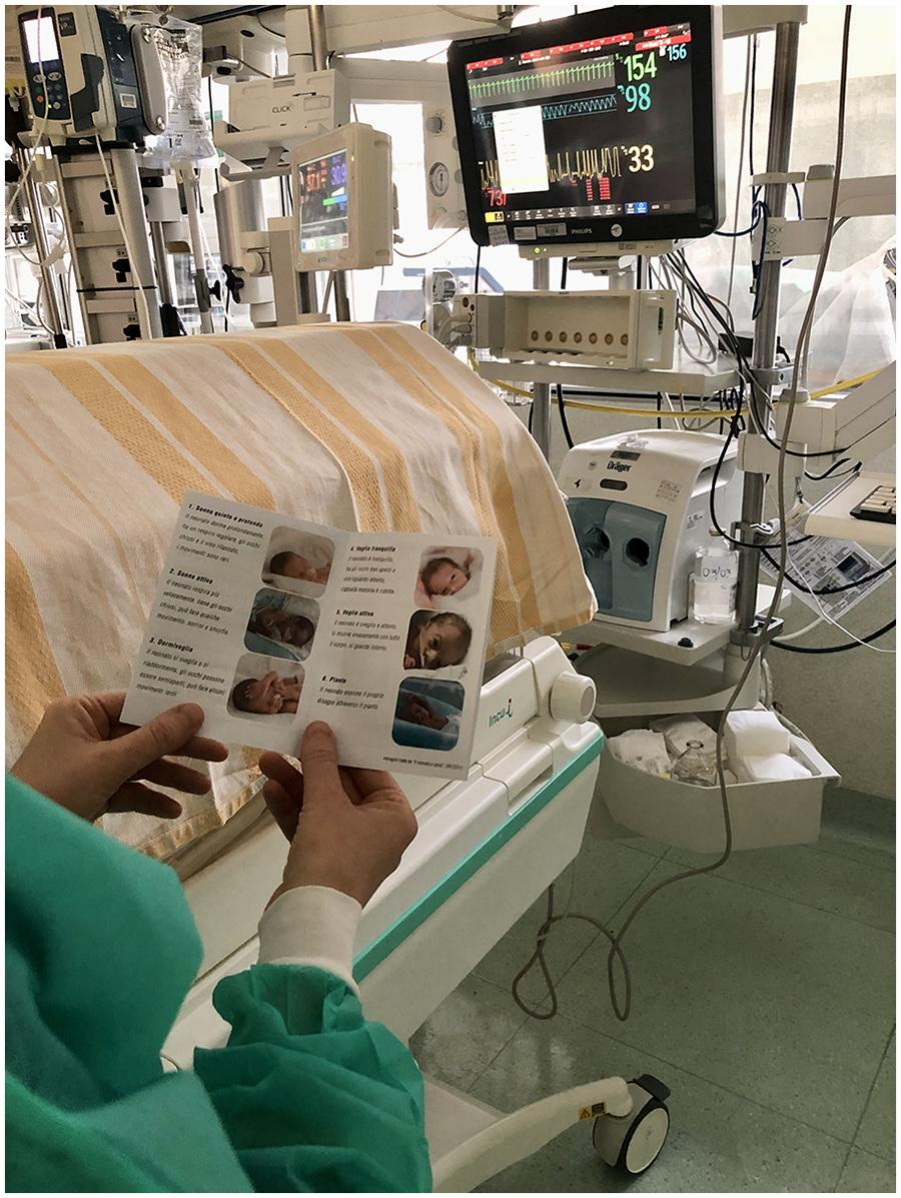
Parent holding one of the educational leaflets during the NICU stay.

The leaflets, as described, were shared with 104 families of preterm children (Figure 3), with a gestational age of 29.95 ± 2.11 (median ± SD) weeks. Among the 104 families, 8 included parents who were not native Italian speakers but had a good comprehension of the Italian language (as required by the study's inclusion criteria), and 18 included parents of mixed nationality (with one parent of non-Italian origin). Median maternal and paternal ages were 34 and 37 years, respectively. The family Socio-Economic Status (SES) index, calculated and classified according to Hollingshead's criteria (46), was 50.57 ± 11.19 (median ± SD). The informal feedback received from parents involved in the project, both during the educational encounters and via email, highlighted the strong emotional resonance of the NICU experience and the perceived value of the materials. Here we report some quotes: “*What memories, seeing them* [the babies] *so tiny and helpless”*, “*Thank you! Now we're big kids, thanks to your invaluable help!”*, “*They* [the leaflets] *are beautiful! I even got emotional seeing her so tiny again! I hope they can help others the way they helped us, and that your project continues to grow”*. Some families suggested that a digital format might be more practical than the paper-based leaflets.

The neurodevelopmental therapists involved in the EI program (one academic researcher and two senior neurodevelopmental therapists) reported, through focus groups held during and after the introduction of the materials, that the use of the leaflets helped in staggering the topics of the parental training in a more coherent and linear manner. They also shared the perception that the educational materials made the EI program more visible and recognizable, fostering collaboration with other healthcare professionals involved in the care of the preterm newborns, such as NICU nurses and physicians. For instance, reviewing the leaflets (collected in a dedicated envelope kept in or near the incubator) together with parents at the bedside created opportunities for shared discussions among the EI team and other healthcare professionals. The therapists further observed that the materials appeared to be better received by families who were already actively engaged in the EI program, while their role as a tool to foster compliance in less engaged families remains unclear.

## 4 Discussion

This paper presents written educational resources and their development within the framework of an EI program for preterm families aimed at supporting parent-infant interaction during NICU stay.

The design process outlined here could serve as a guide for implementing similar tools in EI settings, to foster early parenting skills and promote parental participation, which is known to be relevant for infants' neurodevelopment (47).

Øberg et al. (27) discuss how positive outcomes in EI programs are closely linked to the quality of mutual interaction between the parent and the infant, supported by a trusted therapist. Although challenging, parents' meaningful involvement can be fostered through hands-on caregiving and bodily interaction, which enhances parental confidence and empowerment and reinforces the parent–infant bond, as previously highlighted by Adama et al. (26).

Given this, one of the main strengths of the presented materials lies in the practical guidance provided by the leaflets, which, by offering detailed descriptions of developmentally sensitive interactions while allowing real-time tailoring to the infant's condition, may have facilitated parents' embodied participation during NICU stay. Furthermore, the gradual delivery of information, introduced progressively in line with parents' ongoing observations of their infant, was intended to prevent families from being overwhelmed by excessive input all at once (23). These features may represent a key element in promoting individualized care within routine clinical practice and fostering parental engagement.

An additional strength was the use of a written format, which enabled parents to revisit the content at their own pace, thus supporting understanding and retention over time. The material was designed to facilitate the consolidation of learning over time and to support the continuity and the progressive internalization of key messages (32). Accordingly, during each session the core content from the previous one was reviewed and re-elaborated. This aspect was particularly valuable when only one parent – often the mother – was able to attend the training sessions, mainly due to the limited support provided by current parental leave policies. In the Italian context, fathers are generally entitled to a very limited paternity leave, and this challenge becomes even more evident in the case of preterm birth (48), resulting in an imbalance in parents' participation in the care of their newborns in the NICU (49). The availability of written tools was thus intended to enhance the father's involvement, encouraging active participation in the intervention. As emphasized by Ocampo et al. (50) and Baldoni et al. (51), to create supportive opportunities for NICU fathers is of utmost importance to address their specific needs and consequently have a positive impact on the entire family unit.

Moreover, the low cost of the educational materials provided to parents, consisting of printable brochures, allows for easy reproduction and distribution across different settings.

From clinicians' perspective, the structured organization of topics was perceived by NICU therapists as supporting a more effective delivery of the intervention, enabling easier replication and administration between multiple health care professionals and ensuring consistency and accuracy in the information provided to parents, consistently with Labrie et al. (32) and Bansal et al. (52).

Unlike other interventions such as the SENSE program (33), which provide structured guidelines regarding the type, intensity, and duration of sensory exposures in the NICU, our approach prioritized flexibility, allowing adjustments based on each newborn's clinical conditions and family's specific characteristics. As pointed out by De Rouck and Leys (24), parents in the NICU have evolving informational and emotional needs, mainly depending on their infant's medical trajectory. Therefore, healthcare professionals should adapt the type and mode of information delivery accordingly.

Considering recent evidence on the importance of inclusive language and communication, revising the materials to adopt gender-neutral and inclusive terminology represented a meaningful step toward acknowledging the diversity of family structures and experiences. Although no same-sex parents were present in our population, the language and pictures used to describe parents' and infants' roles was intended to reflect a wide range of different family structures and ethnic groups (34, 53).

Despite these strengths, as this work is primarily descriptive in nature and lacks structured qualitative or quantitative assessment tools, methodological limitations should be acknowledged. First, parents were involved in the design phase of the leaflets through an unstructured method, and this could have limited the identification of families' actual needs and perspectives. In this regard, Guez-Barber et al. (54) and Klawetter et al. (55) emphasize the urgency of a paradigm shift, from viewing patients' families as mere recipients to recognizing them as co-designers of research and care initiatives aimed at creating a positive and nurturing environment.

Moreover, the group of professionals included in the elaboration of the educational resources was relatively small; however, the different backgrounds and levels of expertise in the field allowed for a multidisciplinary discussion of substantial importance (56).

Another area for improvement concerns the collection of feedback from both therapists and parents. The unstructured nature of the feedback collection represents a methodological limitation that may have introduced bias: for parents, it cannot be considered representative of the views of all participating families, with a potential overrepresentation of positive experiences; for therapists, their feedback should be regarded as preliminary qualitative observations rather than measured outcomes. As they were involved in both the development of the leaflets and the EI program, their perceptions may reflect an overestimation of benefits and should therefore be interpreted with caution. Future implementations should therefore employ validated feedback instruments, systematic data collection procedures, and a pre-post or controlled design to enable rigorous evaluation of the material's clarity, relevance and usability, as well as its impact on parental engagement. This would allow for a more comprehensive understanding of the material's strengths and areas for refinement.

Finally, the format of the materials – primarily printed – raises important considerations about accessibility and usability. Given the growing use of digital tools into daily life, it may be worthwhile to explore the integration of technologies – such as a dedicated app – in future versions of the program.

## 5 Conclusions

This study provides a set of educational materials for parents of preterm infants, developed to support their active role in the NICU and promote dyadic interactions with their newborns within an EI program. By offering practical, developmentally sensitive guidance, these materials may strengthen parental engagement; however, adherence to the EI program is influenced by multiple factors beyond the materials themselves, including infant and family characteristics, and the extent to which the leaflets may foster engagement in less compliant families remains to be established.

Although this work offers new insights to enhance EI practices in NICU settings within the broader framework of infant- and family-centered care, further research is needed to validate the effectiveness of this kind of resources and assess their impact on parent-infant outcomes.

## Data Availability

The original contributions presented in the study are included in the article/Supplementary Material, further inquiries can be directed to the corresponding author.

## Supplementary material

Data Sheet1 Stress and Availability Signs - ITA.

Data Sheet 2 Behavioral States - ITA.

Data Sheet 3 Positioning - ITA.

Data Sheet 4 Voice Listening - ITA.

Data Sheet 5 Infant Massage - ITA.

Data Sheet 6 Visual Interaction - ITA.

Data Sheet 7 Finally...Back Home! - ITA.

Data Sheet 8 Stress and Availability Signs - ENG.

Data Sheet 9 Behavioral States - ENG.

Data Sheet 10 Positioning - ENG.

Data Sheet 11 Voice Listening - ENG.

Data Sheet 12 Infant Massage - ENG.

Data Sheet 13 Visual Interaction - ENG.

Data Sheet 14 Finally...Back Home! - ENG.
