# Supplementary material for: Educational materials to empower parents of preterm infants within a family-centered early intervention in the NICU
Source: Front Pediatr. 2026 Jun 9;14:1823643. doi: 10.3389/fped.2026.1823643 (PMC13287061; doi:10.3389/fped.2026.1823643)
Supplement: Data Sheet1 — Stress and Availability Signs - ITA. [file Datasheet1.pdf]

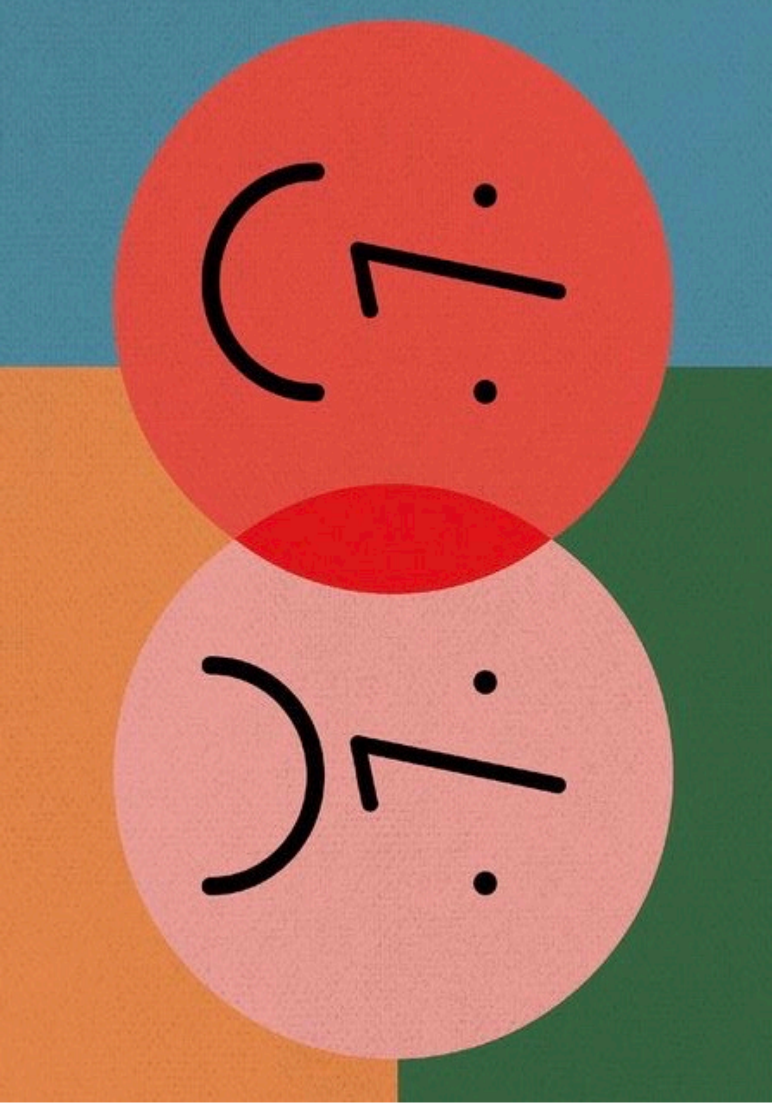

Bonfanti C, Fontana C, et al.

INTERVENTO PRECOCE

SEGNALI DI STRESS E DI DISPONIBILITÀ

NICU, Fondazione IRCCS Ca' Granda
Ospedale Maggiore Policlinico, Milan, Italy

IL LINGUAGGIO DEL VOSTRO BAMBINO

I **segnali di stress e di disponibilità** costituiscono il **linguaggio** che ogni neonato, nella sua individualità, utilizza per **comunicare.**

SEGNALI DI DISPONIBILITÀ

- Indicano stabilità e benessere
- Rappresentano il momento ideale per l'interazione

SEGNALI DI STRESS

- Indicano una disorganizzazione e difficoltà di autoregolazione
- Suggeriscono che il tuo bambino ha bisogno di aiuto per favorire la stabilità e l'interazione

CO-REGOLAZIONE

L'**osservazione** e l'**interpretazione** di tali segnali sono lo strumento per **comprendere i bisogni** del vostro bambino. La co-regolazione è un processo in cui **voi giocate un ruolo fondamentale!**

- ☑ Un occhio sempre attento ai **segnali comportamentali.**
- ☑ Un'attenzione alle possibili **fonti di stress ambientale** (luce, suoni, temperatura…).
- ☑ Rispondete cercando di **modulare l'ambiente** per promuovere la stabilità del vostro bambino.

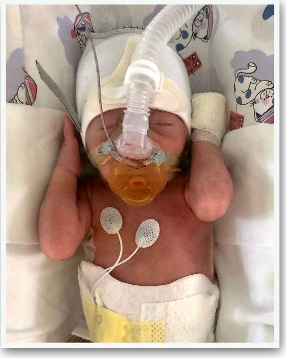

PRINCIPALI CARATTERISTICHE DEI SEGNALI DI DISPONIBILITÀ E DI STRESS

SEGNALI DI DISPONIBILITÀ

- **Stabilità** dei **parametri vitali.**
- Colorito **roseo.**
- **Espressione rilassata** del volto.
- **Mani al viso e alla bocca.**
- **Postura in flessione,** con braccia e gambe raccolte vicino al corpo.
- **Movimenti armonici**.

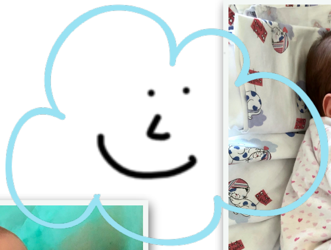
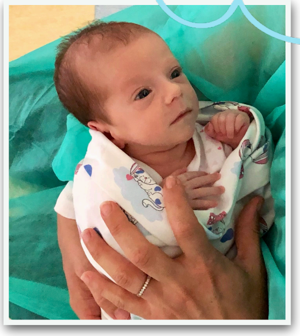
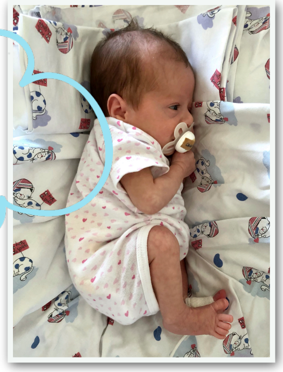

"Sto bene!"

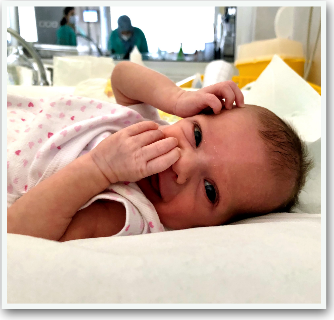

SEGNALI DI STRESS

- **Cambiamenti nei parametri vitali**: respiro irregolare, episodi di desaturazione, aumento o diminuzione del battito cardiaco.
- **Cambiamenti del colorito della pelle**: pallore, marezzatura, rossore.
- **Singhiozzo**, **sbadigli**, rigurgito, spinte.
- Presenza di **tremori** e startle.
- **Segnali motori**: movimenti in estensione di braccia e gambe e schiena inarcata, apertura improvvisa delle mani, movimenti bruschi.
- Tono muscolare ridotto, flaccidità.
- Espressione del viso affaticata con **smorfie**, sguardo in iperallerta.

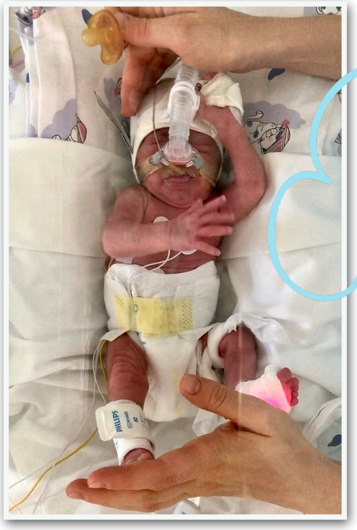
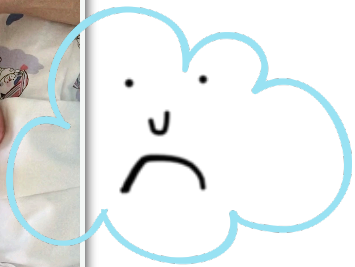

"Mi serve una pausa!"

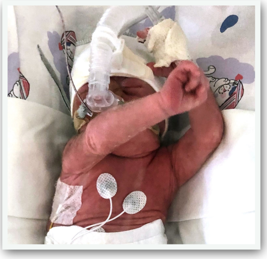
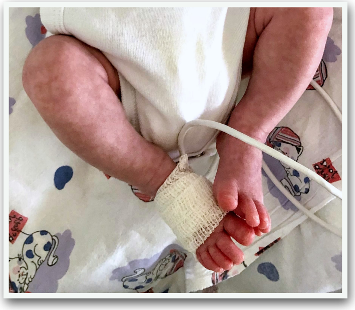

Un grazie speciale ai bambini e ai genitori che ci hanno aiutato in questo progetto!
