# Supplementary material for: Educational materials to empower parents of preterm infants within a family-centered early intervention in the NICU
Source: Front Pediatr. 2026 Jun 9;14:1823643. doi: 10.3389/fped.2026.1823643 (PMC13287061; doi:10.3389/fped.2026.1823643)
Supplement: Data Sheet 2 — Behavioral States - ITA. [file Datasheet2.pdf]

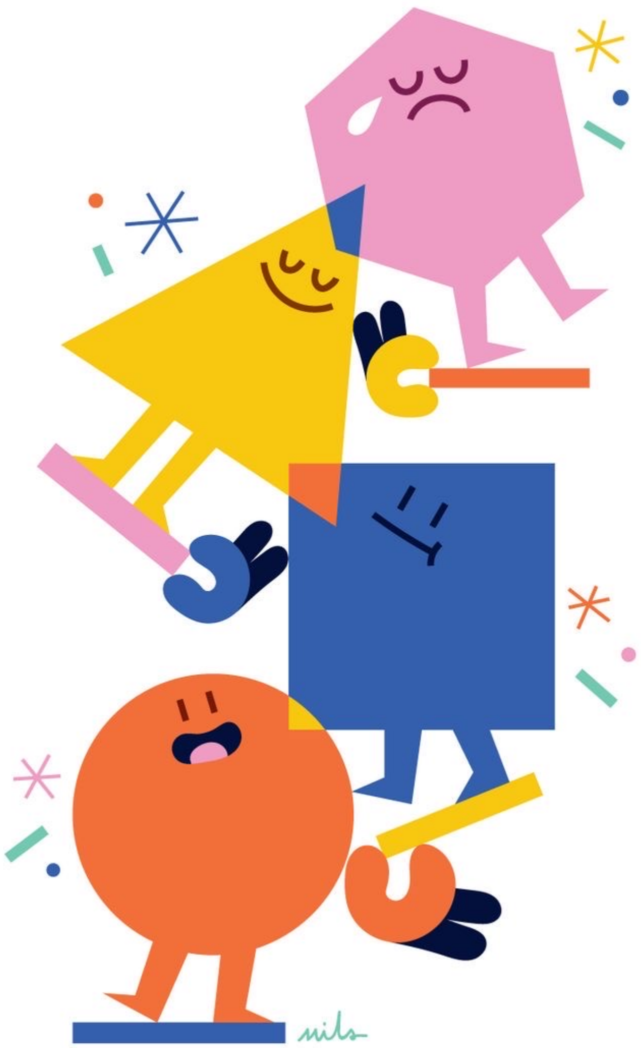

Bonfanti C, Fontana C, et al.

INTERVENTO PRECOCE

STATI COMPORTAMENTALI

NICU, Fondazione IRCCS Ca' Granda
Ospedale Maggiore Policlinico, Milan, Italy

1. Sonno profondo

Il neonato dorme profondamente, ha un respiro regolare, gli occhi chiusi e il viso rilassato, i movimenti sono rari.

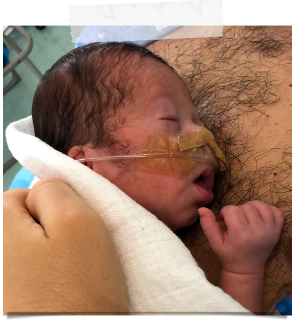

2. Sonno attivo

Il neonato tiene ancora gli occhi chiusi ma respira più velocemente e può fare qualche movimento, sorrisi e smorfie.

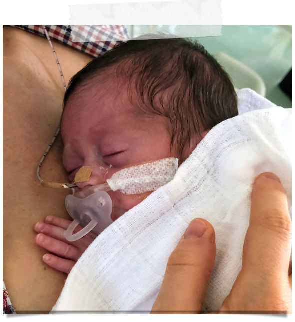

3. Dormiveglia

Il neonato si sveglia e si riaddormenta, gli occhi possono essere chiusi o aperti, può fare alcuni movimenti lenti.

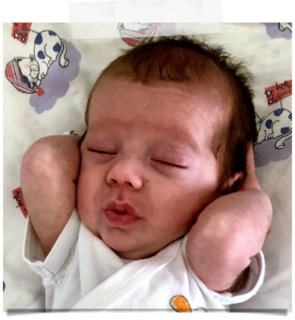

4. Veglia tranquilla

Il neonato è tranquillo, ha gli occhi ben aperti e uno sguardo attento, l'attività motoria è ridotta.

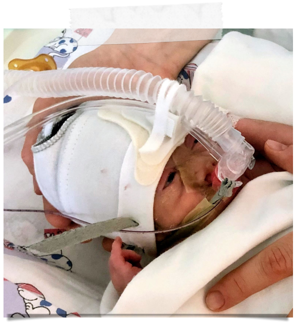

5. Veglia attiva

Il neonato è sveglio e attento, si muove vivacemente con tutto il corpo, si guarda intorno.

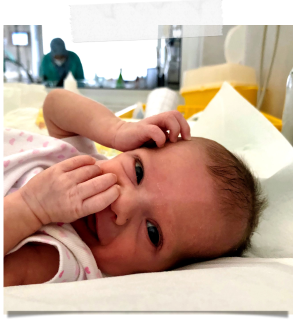

6. Pianto

Il neonato esprime il proprio disagio attraverso il pianto.

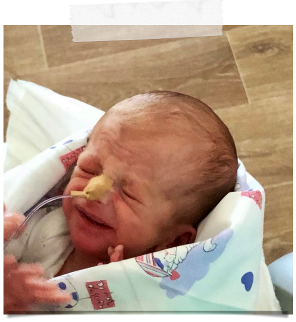

Principali caratteristiche degli Stati Comportamentali

GLI STATI COMPORTAMENTALI DEL NEONATO PRETERMINE

Il neonato pretermine attraversa una **graduale organizzazione** degli stati comportamentali.
All'inizio questi sono **poco differenziati** tra loro, per poi diventare sempre più **chiari e riconoscibili**.

Con il miglioramento dell'organizzazione neurocomportamentale, il tempo del sonno diminuisce e **aumenta la durata dei periodi di veglia,** insieme ad una maggiore **capacità di autoregolazione.**

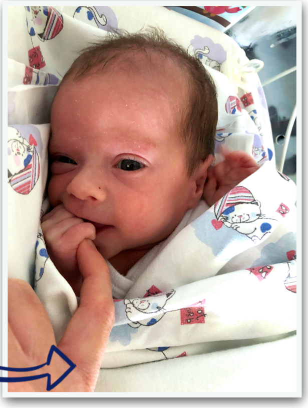

QUANDO INTERAGIRE

Il momento migliore per interagire con il neonato è lo stato di **veglia tranquilla o attiva**, quando manifesta disponibilità e reattività alle esperienze proposte.

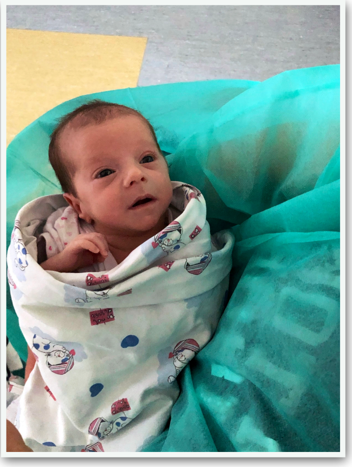

SEGNALI DI INSTABILITÀ COMPORTAMENTALE

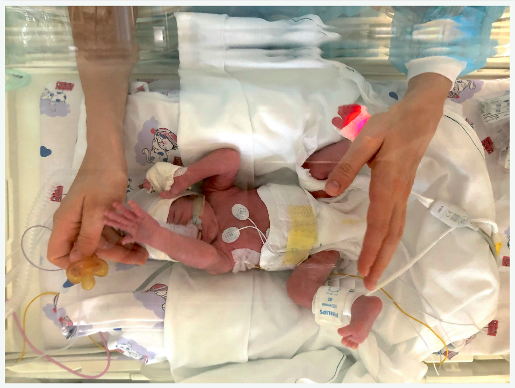

Quando il neonato mostra segnali di instabilità, **ci sta comunicando che ha qualche difficoltà** e che ha bisogno di una **pausa** per riposare e recuperare una maggiore stabilità.
Voi siete i principali **co-regolatori** del vostro bambino e potete **modulare le sue esperienze sensoriali,** promuovendo così il suo sviluppo.

COME AIUTARE IL VOSTRO BAMBINO

- Modulare l'**ambiente** (luce, rumori, suoni…)
- Usare la vostra **voce**
- Tenere delicatamente le sue mani e/o i suoi piedi in una **posizione raccolta** e offrire **un confine con le vostre mani,** usando un **tocco fermo e rassicurante**
- Lasciare che **afferri il vostro dito** ed eventualmente aiutarlo/a ad avvicinarlo alla bocca
- Modificare la **postura**
- Se possibile, **prenderlo/a in braccio**
- Avvolgerlo/a con il **wrapping**

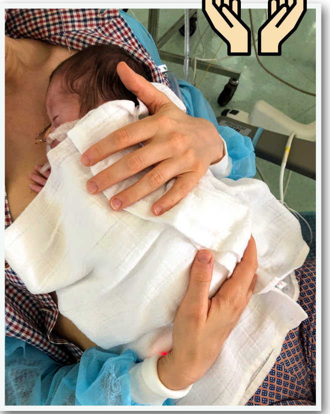

Un grazie speciale ai bambini e ai genitori che ci hanno aiutato in questo progetto!
