# Supplementary material for: Educational materials to empower parents of preterm infants within a family-centered early intervention in the NICU
Source: Front Pediatr. 2026 Jun 9;14:1823643. doi: 10.3389/fped.2026.1823643 (PMC13287061; doi:10.3389/fped.2026.1823643)
Supplement: Data Sheet 3 — Positioning - ITA. [file Datasheet3.pdf]

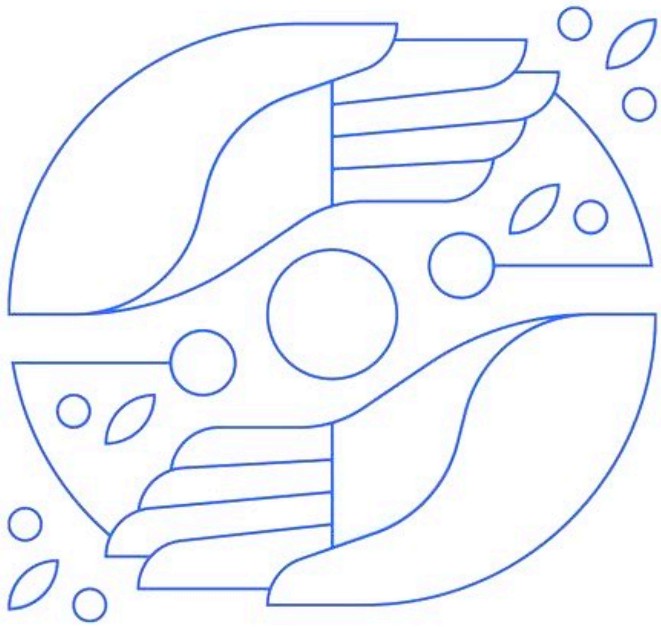

Bonfanti C, Fontana C, et al.

INTERVENTO PRECOCE

POSTURE

NICU, Fondazione IRCCS Ca' Granda
Ospedale Maggiore Policlinico, Milan, Italy

POSTURA E PREMATURITÀ

Alla nascita, il tono muscolare "fisiologicamente" immaturo e la forza di gravità influenzano la postura del neonato pretermine. Il vostro bambino può apparire **disteso sul materassino**, con la **testa ruotata prevalentemente da un lato** e **movimenti ridotti.**

La cura posturale in TIN rappresenta pertanto una parte fondamentale: aiuta a **favorire la stabilità** del neonato e a **ridurre i segnali di stress.**

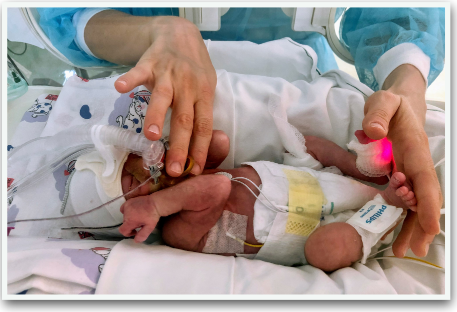

CURA POSTURALE IN TIN

Che cosa è importante?

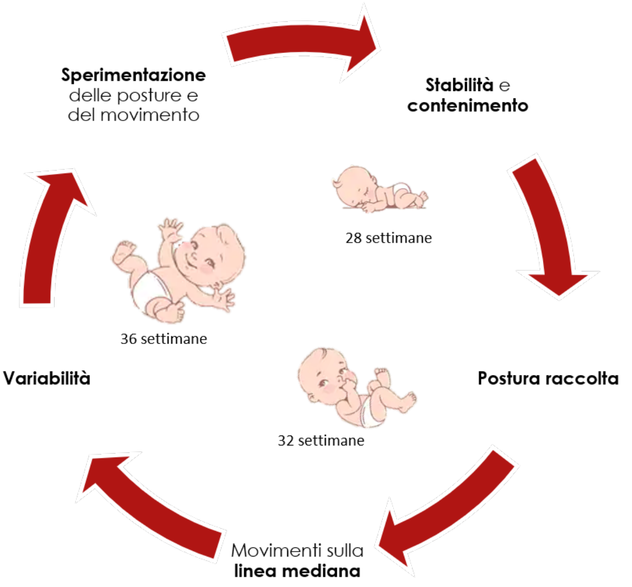

Caratteristiche delle principali posture

POSIZIONE A PANCIA IN SU (SUPINA)

- Consente **libertà di movimento** degli arti.
- Facilita lo scambio e la **relazione** con voi.
- Può causare **maggiore instabilità e difficoltà** perché diventa più difficile portare braccia e gambe verso il centro del corpo.

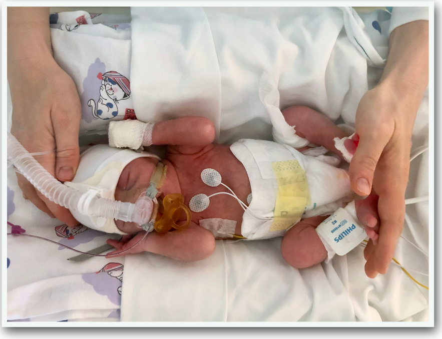

Promuovere una **posizione semiflessa e allineata** (usando ad es. le vostre mani, il nido o un telino), garantendo in questo modo la stabilità.

POSIZIONE A PANCIA IN GIÙ (PRONA)

- Aiuta la **respirazione** e il **battito cardiaco**.
- **Riduce l'instabilità motoria.**
- Quando il bambino è più grande, aiuta a rinforzare la muscolatura che sarà necessaria per il controllo del capo.

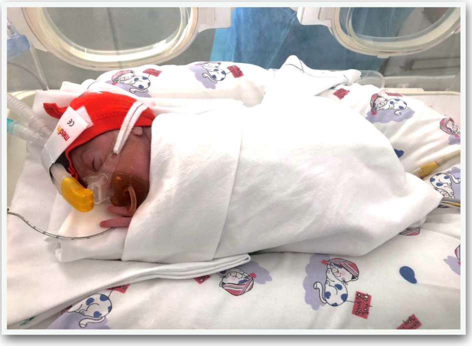

Fate attenzione a porre un **supporto sotto il tronco.**

POSIZIONE SUL FIANCO

- Favorisce la **flessione** e l' **allineamento.**
- Facilita i movimenti **verso il centro del corpo** (ad esempio portare le mani una verso l'altra).
- **Riduce l'instabilità comportamentale.**
- Può facilitare l'alimentazione al biberon.

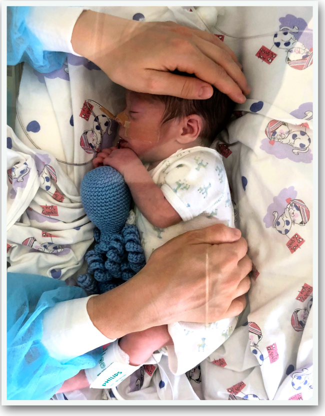

Garantire un **sostegno per la schiena** con il nido e, se necessario, con un piccolo supporto morbido davanti, con **capo e tronco allineati.**

SUPPORTI PER IL CONTENIMENTO POSTURALE

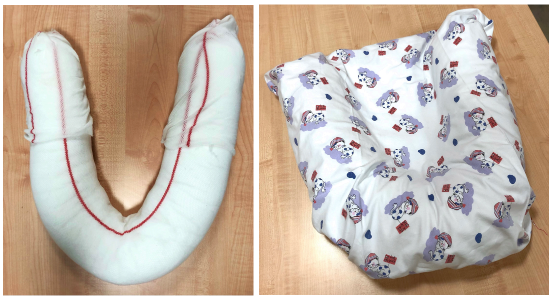

NIDO

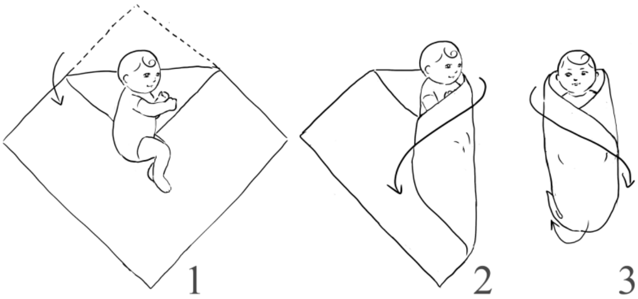

WRAPPING

Un grazie speciale ai bambini e ai genitori che ci hanno aiutato in questo progetto!
