# Supplementary material for: Educational materials to empower parents of preterm infants within a family-centered early intervention in the NICU
Source: Front Pediatr. 2026 Jun 9;14:1823643. doi: 10.3389/fped.2026.1823643 (PMC13287061; doi:10.3389/fped.2026.1823643)
Supplement: Data Sheet 4 — Voice Listening - ITA. [file Datasheet4.pdf]

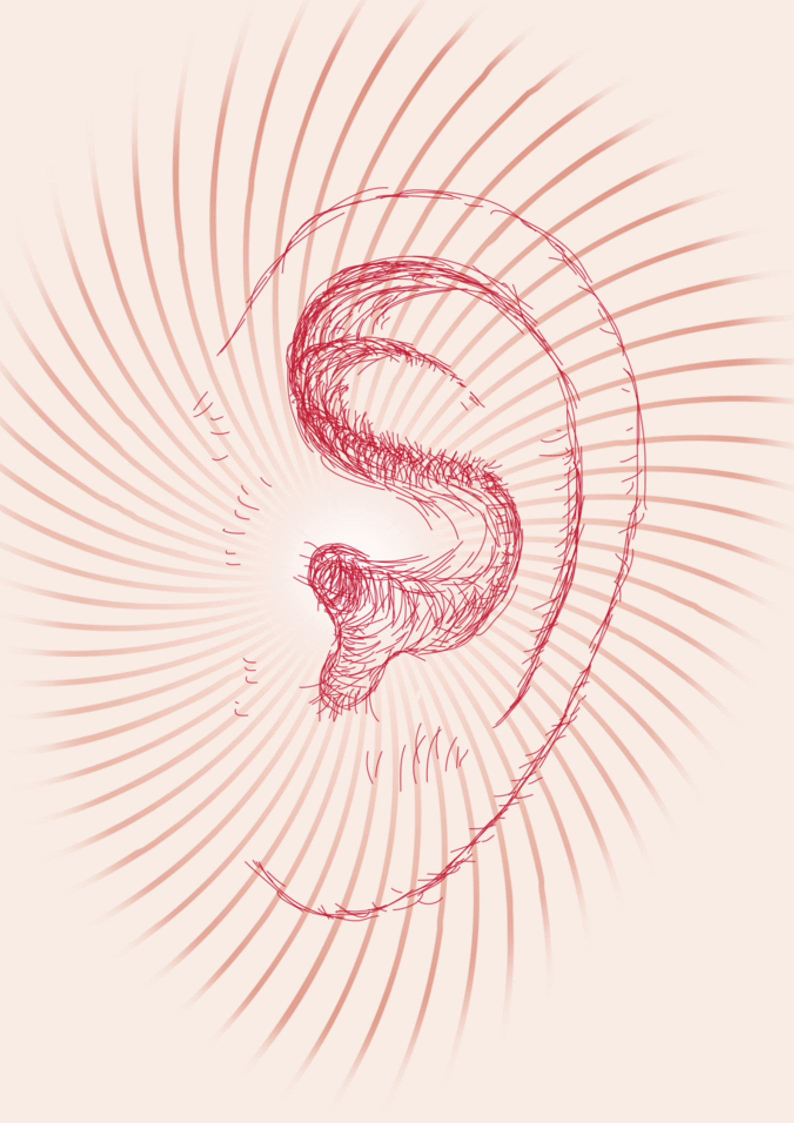

Bonfanti C, Fontana C, et al.

INTERVENTO PRECOCE

ASCOLTO DELLA VOCE

NICU, Fondazione IRCCS Ca' Granda
Ospedale Maggiore Policlinico, Milan, Italy

COME

- All'interno dell'incubatrice, **avvicinando il vostro volto agli oblò**, o quando nel lettino; anche durante la **marsupio** o mentre lo/la tenete **in braccio.**

- Cercate di mantenere l'**ambiente attorno al neonato tranquillo**, regolando, per quanto possibile, le **fonti di rumore** vicine alla culla.

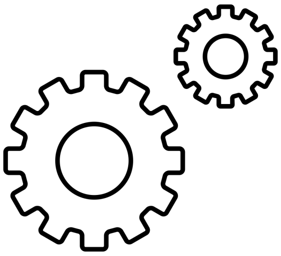

QUANDO

- In uno **stato di veglia tranquilla o attiva**, in presenza di segnali comportamentali di stabilità, o **per favorire il passaggio da uno stato comportamentale all'altro.**

- La vostra voce può anche essere utilizzata quando il vostro bambino sta **piangendo** o **mostrando segnali di stress,** per aiutarlo/a a ritrovare una maggiore regolazione.

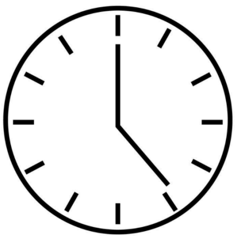

MODALITÀ CON CUI FAVORIRE LE ESPERIENZE UDITIVE E MULTISENSORIALI ATTRAVERSO L'ASCOLTO DELLA VOCE PER LA PROMOZIONE DEL NEUROSVILUPPO

PROPOSTE

La vostra voce è il suono preferito del neonato!

- **Parlate** gentilmente al vostro bambino.
- **Leggete** un libro o raccontate una storia.
- Usate la vostra voce per **creare una melodia,** utilizzando solo alcuni suoni modulati.
- **Cantate** una ninna nanna o una canzone.

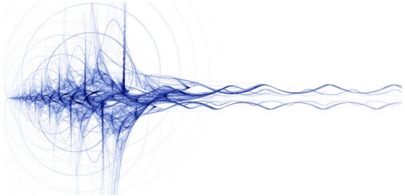

Nel fare queste proposte, cercate di **modulare la vostra voce,** mantenendo un **volume basso e un tono tranquillo**.

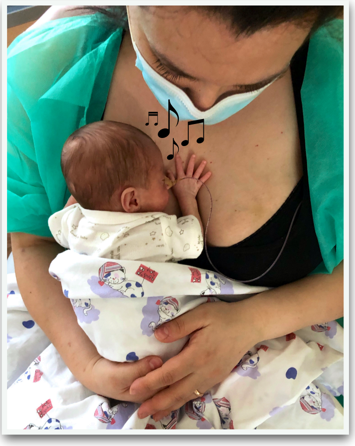

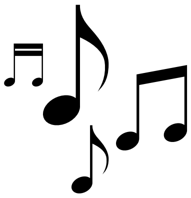

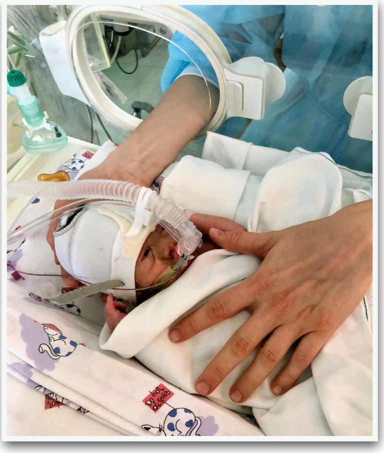

- L'uso della voce per parlare al vostro bambino può iniziare anche molto precocemente, come modalità di contenimento **quando non è ancora possibile toccarlo/a.**

- Le esperienze appena descritte possono accompagnare altri tipi di proposte, in particolare le **attività di cura quotidiana.**

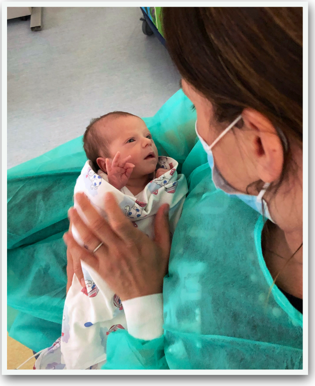

ALCUNE ATTENZIONI

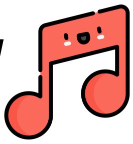

- Mentre parlate o leggete o cantate al vostro bambino, **osservate i suoi segnali** e modulate le proposte in relazione ad essi.

- Cercate di **evitare di fare troppe proposte contemporaneamente.**

- Se il neonato mostra segni di stress:
  - ✓ provate a **ridurre l'intensità** della proposta, ed eventualmente **fate una pausa;**
  - ✓ **aiutatelo/a a riorganizzarsi** (ad es. offrendo un contenimento);
  - ✓ provate a riprendere o, in alternativa, interrompete la proposta.

Un grazie speciale ai bambini e ai genitori che ci hanno aiutato in questo progetto!
