# Supplementary material for: Educational materials to empower parents of preterm infants within a family-centered early intervention in the NICU
Source: Front Pediatr. 2026 Jun 9;14:1823643. doi: 10.3389/fped.2026.1823643 (PMC13287061; doi:10.3389/fped.2026.1823643)
Supplement: Data Sheet 5 — Infant Massage - ITA. [file Datasheet5.pdf]

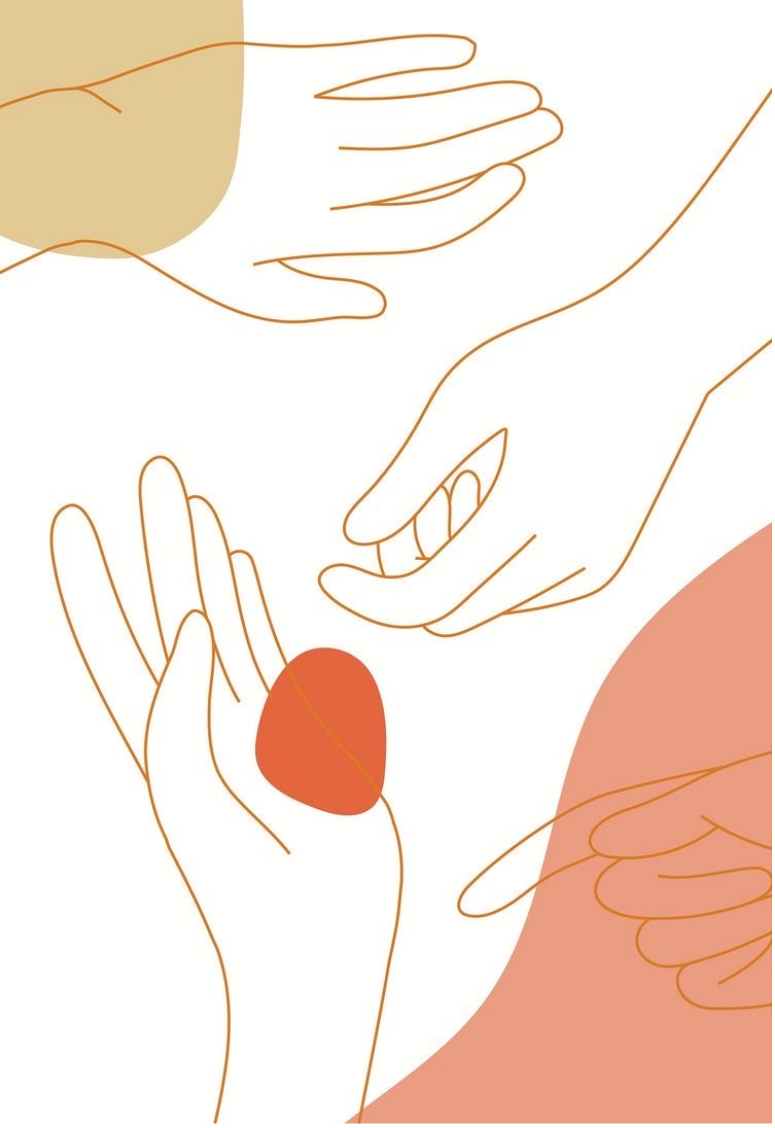

Bonfanti C, Fontana C, et al.

INTERVENTO PRECOCE

MASSAGGIO INFANTILE

NICU, Fondazione IRCCS Ca' Granda
Ospedale Maggiore Policlinico, Milan, Italy

COME

- Prediligere la **posizione prona durante la marsupio**. In alternativa, **sul fianco** all'interno dell'incubatrice o del lettino, oppure in braccio.
- **Pelle nuda** se il bambino mostra di tollerare la proposta, con eventualmente un telino o una copertina o la tutina.

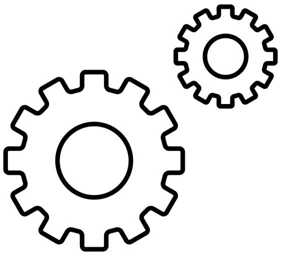

QUANDO

- In condizioni di **stabilità clinica,** a partire da **30 settimane di età post-concezionale.**
- Stato di **veglia tranquilla.**
- **Non troppo vicino alla proposta di massaggio precedente** e, se possibile, **tra un pasto e l'altro.**
- Per **alcuni momenti** nell'arco della giornata, **quando** il bambino **non presenta segnali di stress.**

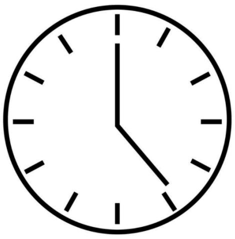

MODALITÀ CON CUI FAVORIRE LE ESPERIENZE TATTILI E MULTISENSORIALI ATTRAVERSO IL MASSAGGIO INFANTILE PER LA PROMOZIONE DEL NEUROSVILUPPO

MASSAGGIO INFANTILE

- Iniziate con un tocco delicato, appoggiando **una mano sulla testa e l'altra sulla schiena** del vostro bambino.
- Tenendo **una mano ferma**, iniziate a **muovere l'altra lentamente.**
- Proseguite con **entrambe le mani**: applicate una **leggera pressione** con il **palmo e le dita,** partendo dalla testa e continuando lungo le spalle, la schiena e le gambe.
- Cercate di mantenere un **ritmo lento e continuo**.
- Terminate la proposta **riducendo gradualmente il massaggio**, fino a far percepire al neonato un tocco fermo e contenitivo.

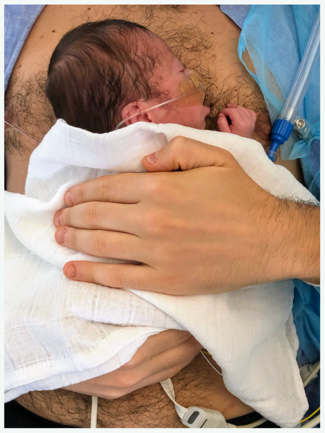

DURANTE LA MARSUPIO

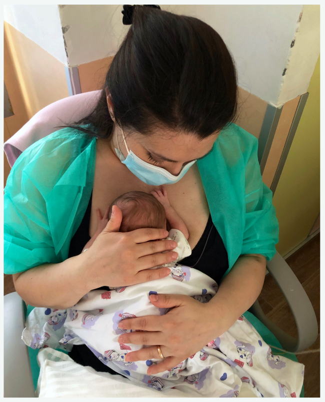

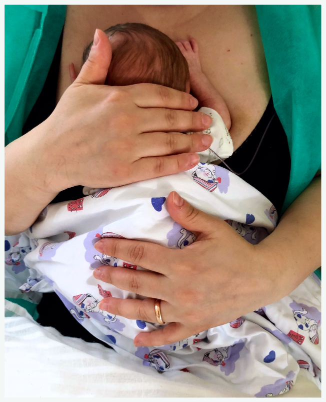

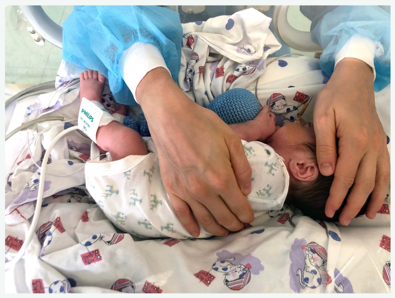

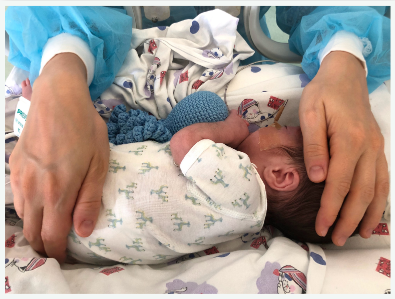

SUL FIANCO

ALCUNE ATTENZIONI

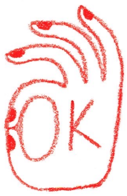

- **Osservate come il bambino risponde alla proposta**: se mostra segnali di stress, potete usare un **tocco fermo e contenitivo** per aiutarlo/a.
- Ponete attenzione alla **stabilità posturale,** offrendo un supporto con il nido e il telino.
- Cercate di iniziare e terminare il massaggio nel modo più **graduale** possibile.
- Durante il massaggio, prestando sempre attenzione ai segnali comportamentali, potete ad esempio usare la **vostra voce per parlare o cantare una melodia.**

Un grazie speciale ai bambini e ai genitori che ci hanno aiutato in questo progetto!
