# Supplementary material for: Educational materials to empower parents of preterm infants within a family-centered early intervention in the NICU
Source: Front Pediatr. 2026 Jun 9;14:1823643. doi: 10.3389/fped.2026.1823643 (PMC13287061; doi:10.3389/fped.2026.1823643)
Supplement: Data Sheet 6 — Visual Interaction - ITA. [file Datasheet6.pdf]

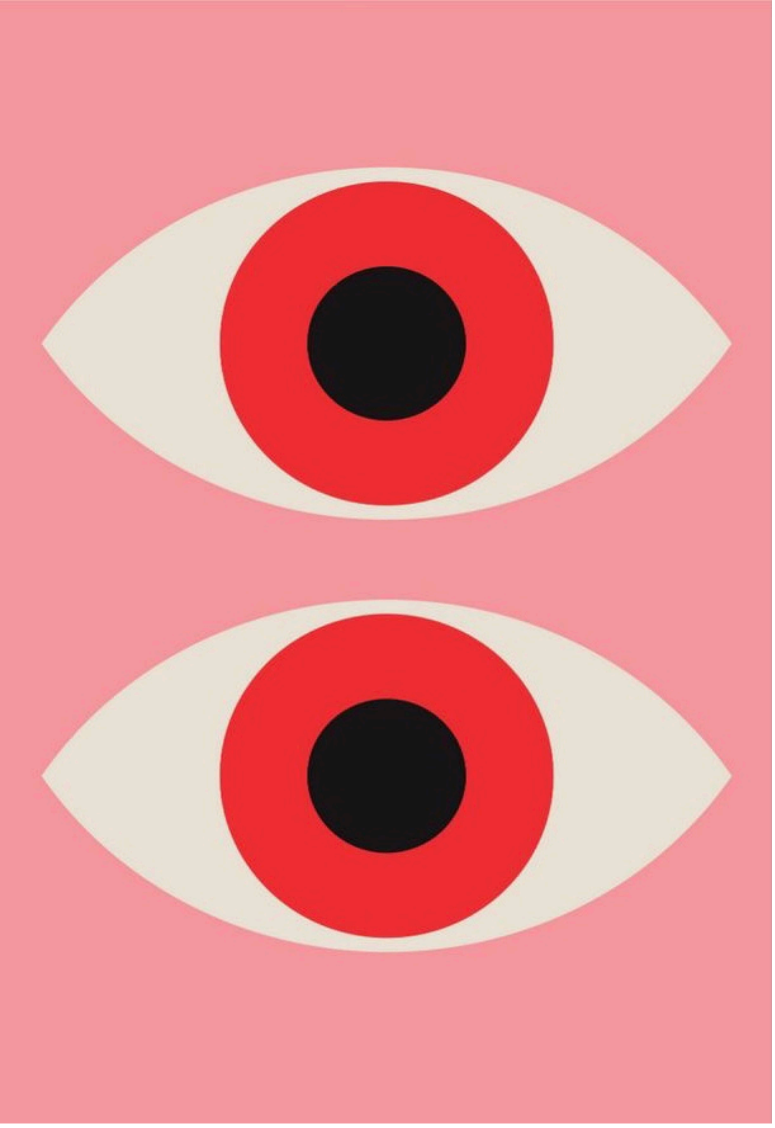

Bonfanti C, Fontana C, et al.

INTERVENTO PRECOCE

INTERAZIONE VISIVA

NICU, Fondazione IRCCS Ca' Granda
Ospedale Maggiore Policlinico, Milan, Italy

COME

- **Posizione a pancia in su**, o in alternativa sul fianco, sulle vostre gambe o nella culla.
- Fonte di **luce posteriore**.
- Offrire un **contenimento** (es. wrapping) ma lasciando il **capo libero di muoversi da entrambe i lati.**

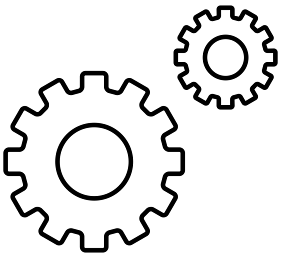

QUANDO

- In condizioni di **stabilità clinica**, a partire da **34 settimane di età post-concezionale.**
- Stato di **veglia tranquilla.**
- **Per alcuni momenti** nell'arco della giornata, quando il bambino è disponibile.

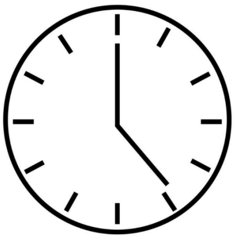

MODALITÀ CON CUI FAVORIRE LE ESPERIENZE VISIVE E MULTISENSORIALI ATTRAVERSO L'INTERAZIONE VISIVA PER LA PROMOZIONE DEL NEUROSVILUPPO

INTERAZIONE DIRETTA TRA IL VOSTRO VOLTO E LO SGUARDO DEL NEONATO

Avvicinate il vostro volto a quello del vostro bambino, restando fermi e **permettendogli di esplorarlo.** Se necessario, cercate di coinvolgerlo utilizzando la vostra **voce**.

Successivamente, potete iniziare a **muovervi lentamente,** invitando il bambino a seguirvi con lo sguardo.

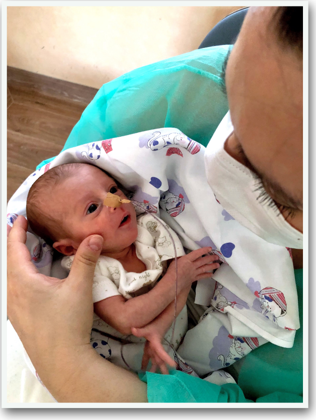

TARGET E OGGETTI AD ALTO CONTRASTO

Proponete al vostro bambino figure, libretti, giochi morbidi **ad alto contrasto** (bianco-nero, rosso-giallo) e **disegni semplici** che catturino la sua attenzione.

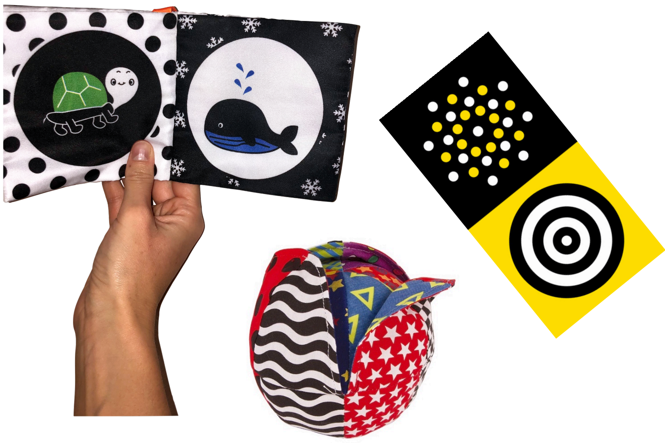

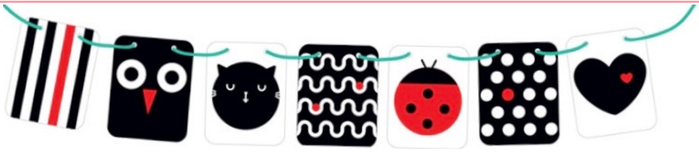

- Abbiate cura di proporre **un oggetto alla volta**, ad una distanza di circa **20-30 cm** dal volto.
- Usate **movimenti lenti**, per aiutare il bambino a seguirvi con lo sguardo.
- Iniziate promuovendo la **fissazione** e solo successivamente l'**inseguimento orizzontale**. Nelle settimane successive, il bambino sarà in grado di seguire anche in verticale e su un arco.

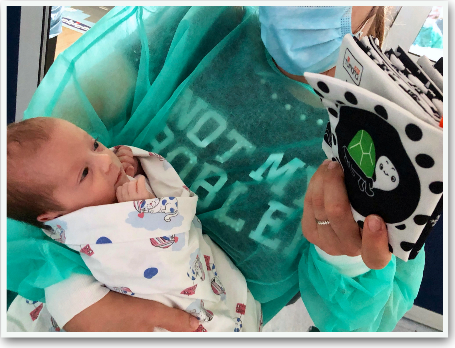

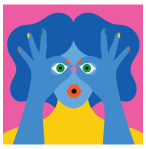

ALCUNE ATTENZIONI

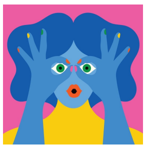

- Osservate i segnali del vostro bambino e cercate di **modulare la proposta** in relazione ad essi.
- Fate attenzione a trovarvi in un **ambiente tranquillo**, con **luce soffusa** (preferibilmente proveniente da dietro), ed eventualmente **proteggete gli occhi del bambino dalle luci dirette** con le mani o con un telino.
- Cercate di garantire una **postura stabile**, per favorire uno stato di veglia tranquilla, l'attenzione e l'interazione. Se necessario, potete aiutarlo/a **aumentando il contenimento** o **sostenendo delicatamente la testa**.

Un grazie speciale ai bambini e ai genitori che ci hanno aiutato in questo progetto!
