# Supplementary material for: Educational materials to empower parents of preterm infants within a family-centered early intervention in the NICU
Source: Front Pediatr. 2026 Jun 9;14:1823643. doi: 10.3389/fped.2026.1823643 (PMC13287061; doi:10.3389/fped.2026.1823643)
Supplement: Data Sheet 7 — Finally...Back Home! - ITA. [file Datasheet7.pdf]

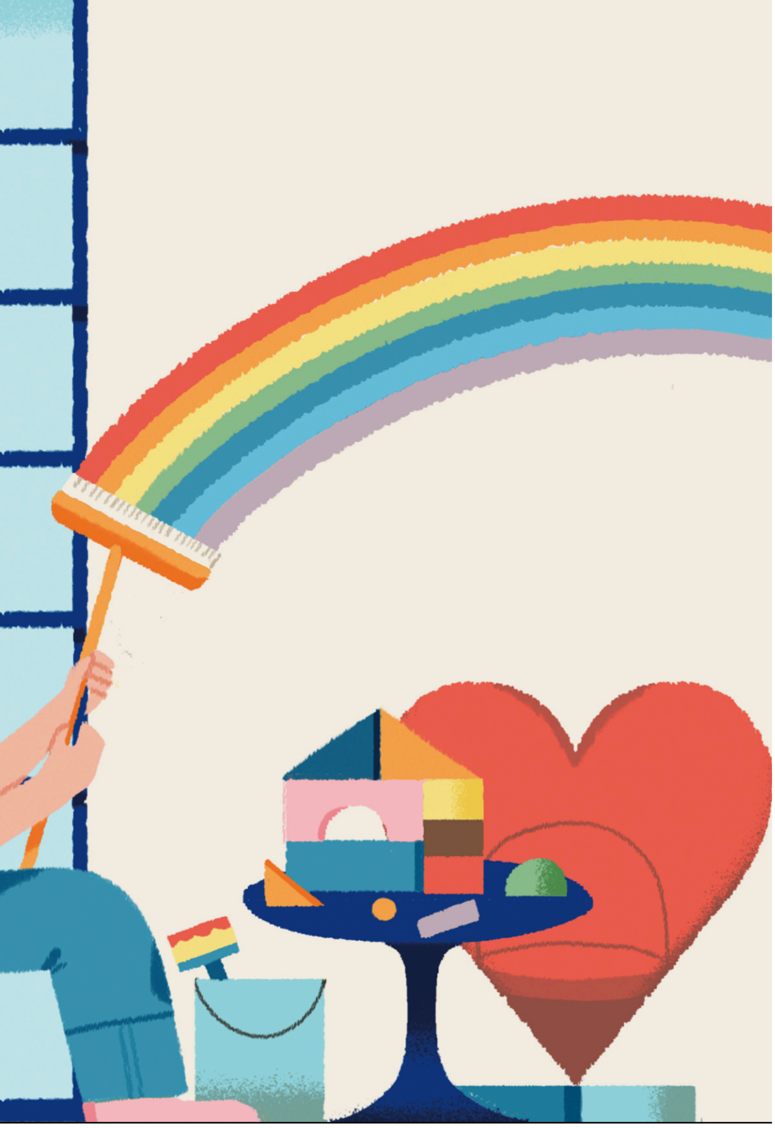

Bonfanti C, Fontana C, et al.

INTERVENTO PRECOCE

FINALMENTE…
A CASA!

NICU, Fondazione IRCCS Ca' Granda
Ospedale Maggiore Policlinico, Milan, Italy

ALCUNI CONSIGLI UTILI

- Prendetevi cura dell'ambiente, cercando di offrire **esperienze sensoriali adeguate**, evitando luci e suoni troppo intensi.

- Aiutate il vostro bambino ad **organizzare il ritmo sonno-veglia**, modulando le diverse proposte nell'arco della giornata in base ai suoi bisogni.

- Durante il giorno, proponete **momenti piacevoli di gioco e di interazione,** promuovendo così l'attenzione e la stabilità del bambino.

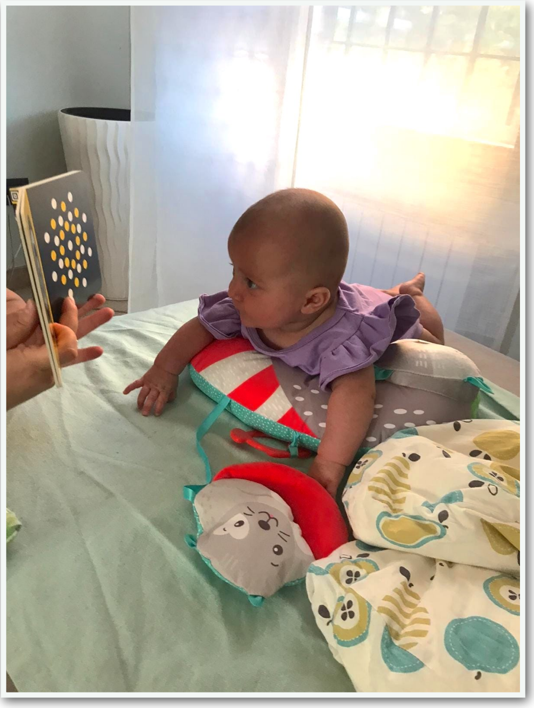

Promuovere il benessere e lo sviluppo del neonato a casa attraverso l'interazione e il gioco

POSTURA E CONTENIMENTO

- Favorire una **postura contenuta** usando il nido e, se necessario, un wrapping leggero.
- **Variare le posture durante la giornata**: pancia in su, pancia in giù e sul fianco. Proporre posizioni diverse anche quando tenete il bambino in braccio.

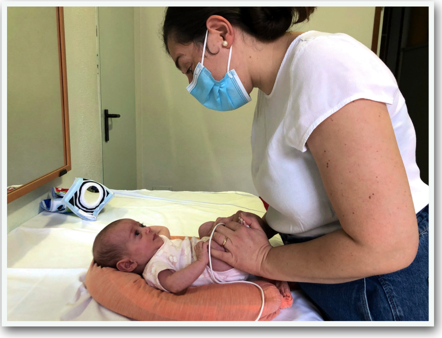

DA SVEGLI!

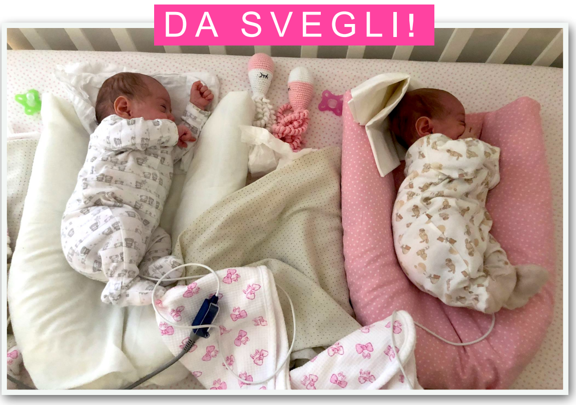

- Favorire **la posizione prona e sul fianco per brevi periodi,** solo quando il bambino è in uno stato di **veglia,** con un sostegno sotto il tronco e utilizzando oggetti ad alto contrasto.
- Offrire **opportunità per sperimentare il movimento** e favorire il **contenimento tra le vostre braccia**.

QUALI ATTIVITÀ POTETE FARE INSIEME?

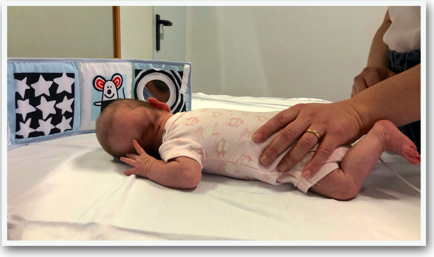

- **Interazione con il vostro volto**, utilizzando lo sguardo e la voce, ad esempio cantando, parlando o leggendo un libro.
- **Esplorazione visiva** con figure, libretti, giochi morbidi **ad alto contrasto** (bianco-nero, giallo-rosso).
- Esperienze di **contatto pelle a pelle** e di **massaggio.**

QUANDO?

- Stato di **veglia tranquilla**.
- Ambiente **calmo e tranquillo**.
- **Per alcuni momenti** nell'arco della giornata, quando il bambino è disponibile.

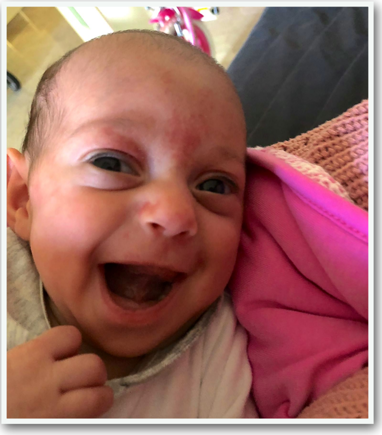

Un grazie speciale ai bambini e ai genitori che ci hanno aiutato in questo progetto!
