# Supplementary material for: Educational materials to empower parents of preterm infants within a family-centered early intervention in the NICU
Source: Front Pediatr. 2026 Jun 9;14:1823643. doi: 10.3389/fped.2026.1823643 (PMC13287061; doi:10.3389/fped.2026.1823643)
Supplement: Data Sheet 8 — Stress and Availability Signs - ENG. [file Datasheet8.pdf]

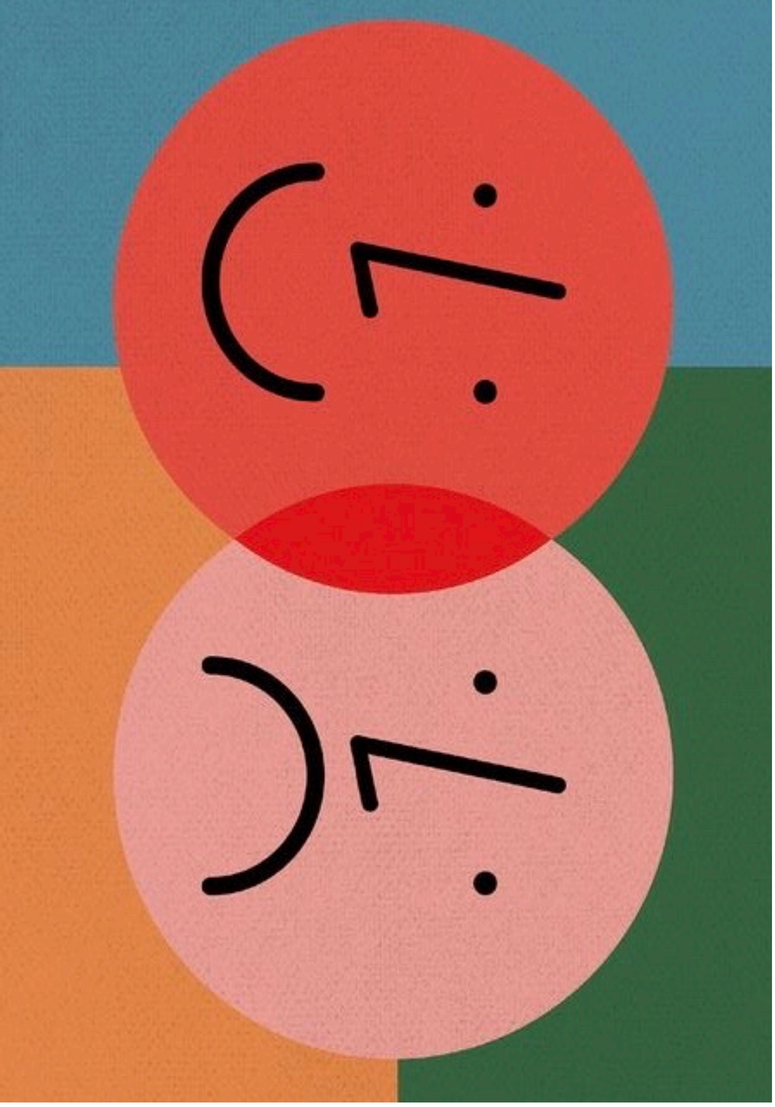

Bonfanti C, Fontana C, et al.

EARLY INTERVENTION

STRESS AND AVAILABILITY SIGNS

NICU, Fondazione IRCCS Ca' Granda
Ospedale Maggiore Policlinico, Milan, Italy

YOUR BABY'S LANGUAGE

Each newborn has his/her own language: **stress and availability cues** are the way through which your baby **communicate** with you and others.

AVAILABILITY SIGNS

- Reflect <u>stability and well-being</u>
- Your baby is <u>ready to interact with you</u>

STRESS SIGNS

- Indicate <u>subsystems' disorganization and difficulty in self-regulation</u>
- Suggest that your baby <u>needs help to promote subsystems' organization and interaction</u>

CO-REGULATION

The **observation** and **interpretation** of these cues helps you to **understand** your child's **individual** needs. **Co-regulation** is a two-way interactive process, in which you play a **fundamental role!**

- ☑ **Look** at your baby's **neurobehavioral signs.**
- ☑ Pay attention to all the possibile **environmental sources of stress** (light, noise, temperature…).
- ☑ **Adjust the environment** to enhance your child's stability.

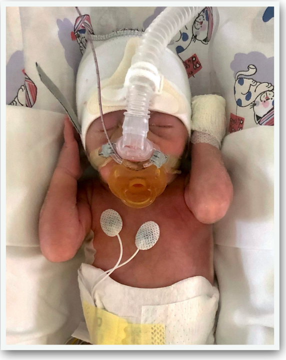

Main Characteristics of Availability and Stress Signals

AVAILABILITY SIGNS

- Physiological **stability.**
- **Healthy skin colour.**
- **Relaxed face**.
- **Hands to face and mouth.**
- **Flexed posture**, with arms and legs close to the body.
- **Smooth** body **movements**.

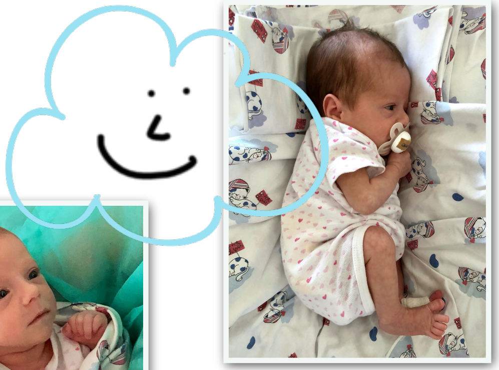

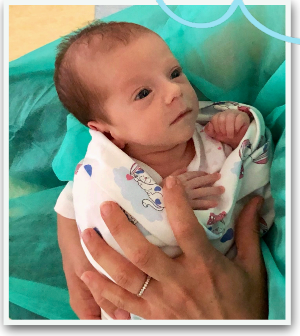

"I'm feeling well!"

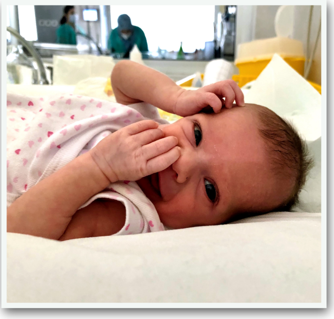

STRESS SIGNS

- **Changes in vital signs**: increasing respiratory rate, decreasing oxygen levels, increasing or decreasing heart rate.
- **Skin colour changes**: pallor, mottling, flushing.
- **Hiccup**, **yawning**, voiding, **straining**.
- **Tremors** and startles.
- **Motor signs**: arms up, legs extension, finger splaying, trunk arching, jerky movements.
- Generalized hypotonia.
- **Face grimaces**, hyper alert or gaze aversion.

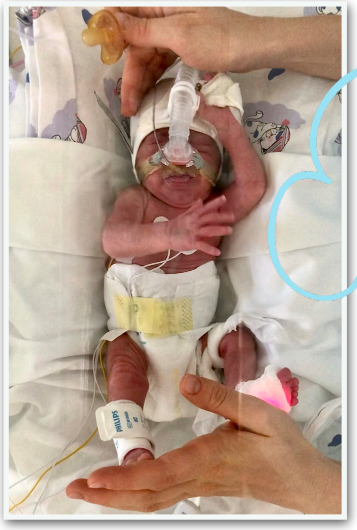

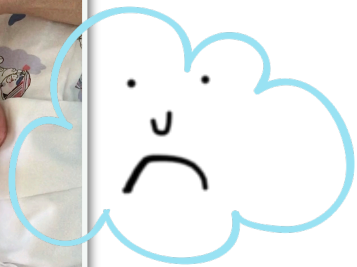

"I need a break!"

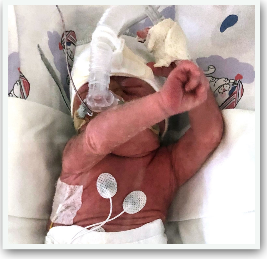

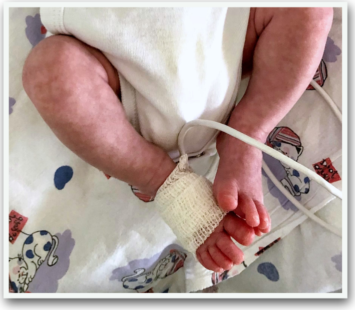

Special thanks to babies and parents that helped us in this project!
