# Supplementary material for: Educational materials to empower parents of preterm infants within a family-centered early intervention in the NICU
Source: Front Pediatr. 2026 Jun 9;14:1823643. doi: 10.3389/fped.2026.1823643 (PMC13287061; doi:10.3389/fped.2026.1823643)
Supplement: Data Sheet 9 — Behavioral States - ENG. [file Datasheet9.pdf]

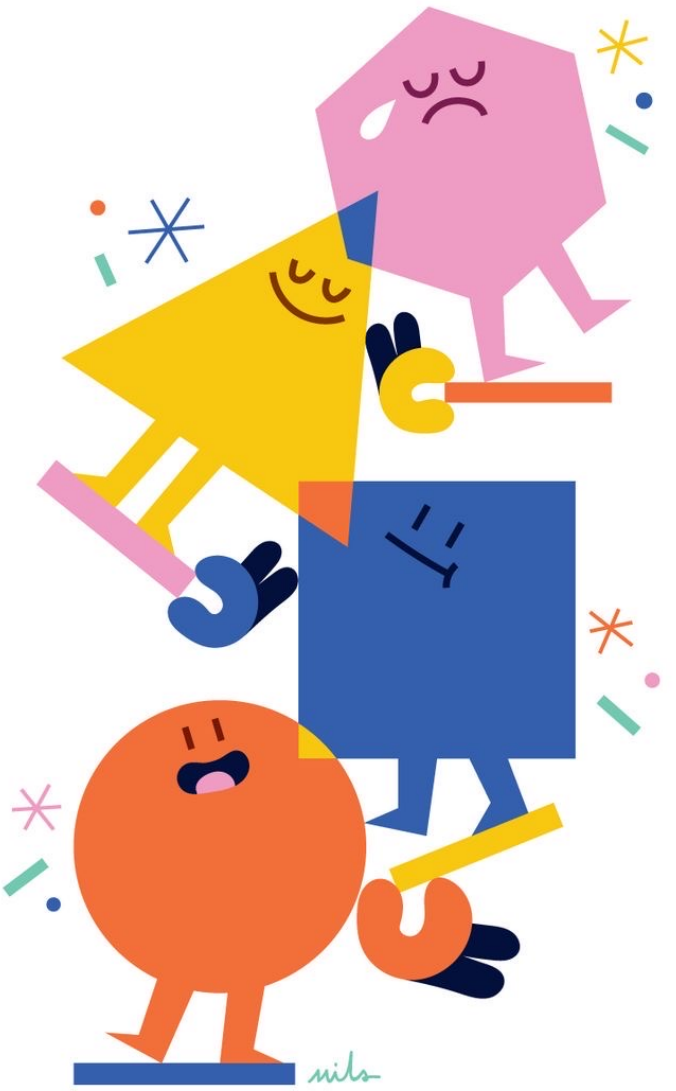

Bonfanti C, Fontana C, et al.

EARLY INTERVENTION

BEHAVIORAL STATES

NICU, Fondazione IRCCS Ca' Granda
Ospedale Maggiore Policlinico, Milan, Italy

1. Deep sleep

The newborn sleeps deeply, breathes regularly, keeps his/her eyes closed and face relaxed, there is no motor activity.

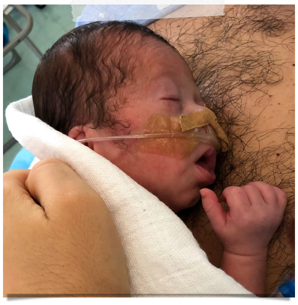

2. Light sleep

The newborn still keeps his/her eyes closed but has faster breathing and can do some random movements or spontaneous smiles or grimaces.

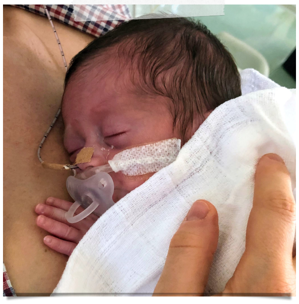

3. Drowsy

The baby is half-asleep, eyes may be open or closed and there could be some slow movements.

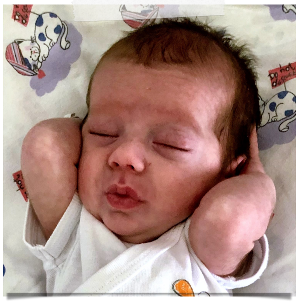

4. Quiet alert

The baby is calm and relaxed, with eyes open and bright look and slow motor activity.

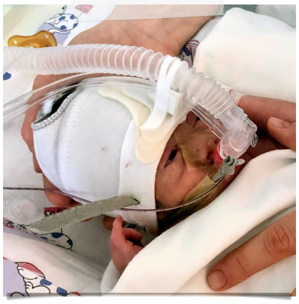

5. Active alert

The infant is clearly awake and aroused, moves lively with the whole body and looks around.

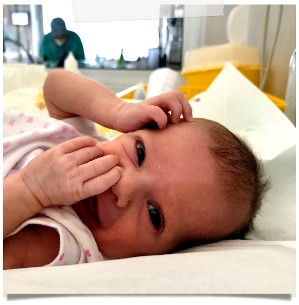

6. Crying

The newborn shows his/her own discomfort through intense crying.

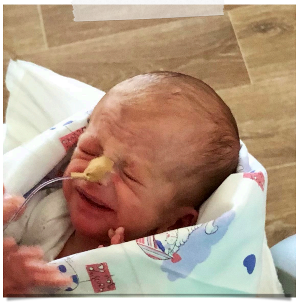

BEHAVIORAL STATES' MAIN CHARACTERISTICS

PRETERM INFANT'S BEHAVIORAL STATES

The preterm infant goes through a ***gradual organization*** *of behavioral states. At the beginning, they are* ***little differentiated****, and then they become more and more* ***clear and defined*** *one from the other. As neurobehavioral organization improves, time spent in sleep decreases and is replaced by increasingly* ***long periods of alertness.***

These changes get along with a higher ***self-regulation ability.***

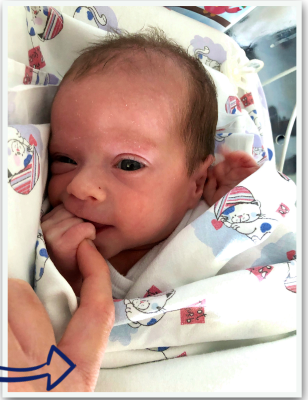

WHEN TO INTERACT

The best moment to interact with your baby is during ***quiet or active states****, when he or she is showing responsiveness to the proposed experiences.*

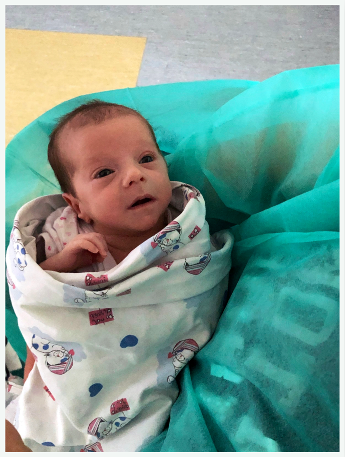

NEUROBEHAVIORAL AVOIDANCE SIGNS

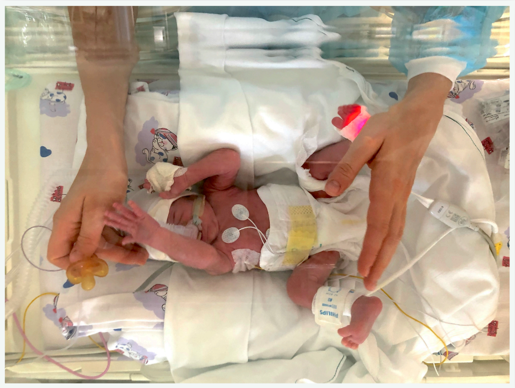

When your baby shows signs of instability, he/she is telling us that he/she is ***having some difficulty*** *and* ***needs a break****.*

You, as the main expert and ***co-regulator*** *of your baby, can help him/her to* ***modulate sensory experiences*** *and* ***support neurodevelopment.***

WHAT YOU CAN DO TO HELP YOUR BABY

- *Adjust the* ***environment*** *(lights, sounds…)*
- *Use your* ***voice***
- *Gently hold your baby in a* ***flexed position*** *and give* ***containment with your hands,*** *using* ***firm and comforting touch***
- *Let your baby* ***hold onto your finger*** *and help him/her bringing it to the mouth*
- *Change his/her* ***position***
- *If possibile, hold him/her* ***skin-to-skin or in your arms***
- *Provide containment through* ***wrapping***

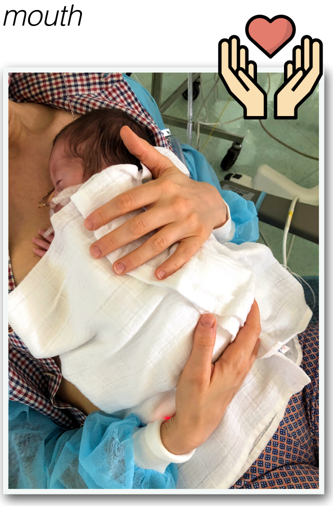

Special thanks to babies and parents that helped us in this project!
