# Supplementary material for: Educational materials to empower parents of preterm infants within a family-centered early intervention in the NICU
Source: Front Pediatr. 2026 Jun 9;14:1823643. doi: 10.3389/fped.2026.1823643 (PMC13287061; doi:10.3389/fped.2026.1823643)
Supplement: Data Sheet 10 — Positioning - ENG. [file Datasheet10.pdf]

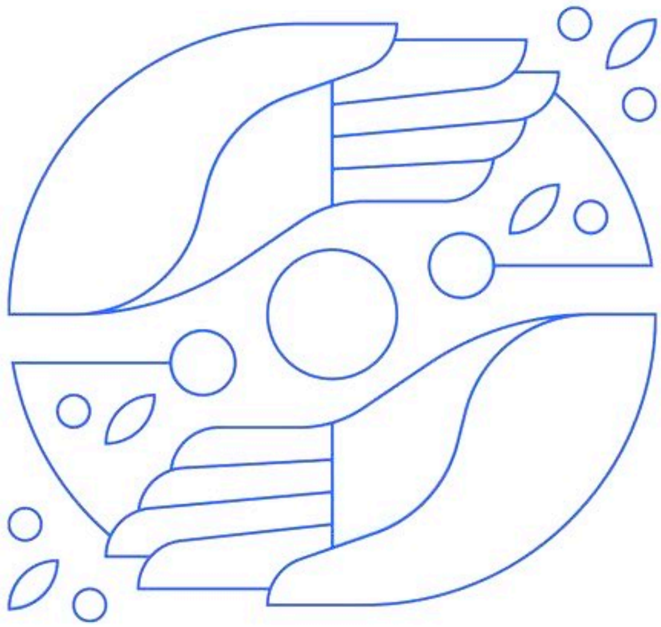

Bonfanti C, Fontana C, et al.

EARLY INTERVENTION

POSITIONING

NICU, Fondazione IRCCS Ca' Granda
Ospedale Maggiore Policlinico, Milan, Italy

POSITIONING AND PREMATURITY

At birth, the neonatal "physiological" hypotonia and the gravity affect preterm infant's posture and ability to reach typical full-term newborn flexed posture and midline orientation. Your baby may appear **flattened** on the mattress, with **head mainly turned on one side** and **reduced movements**.

Positioning is an essential component of the care in the NICU **to promote infant stability and to reduce stress signs.**

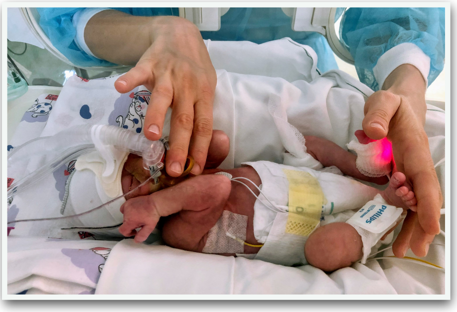

POSITIONING IN THE NICU

What's important?

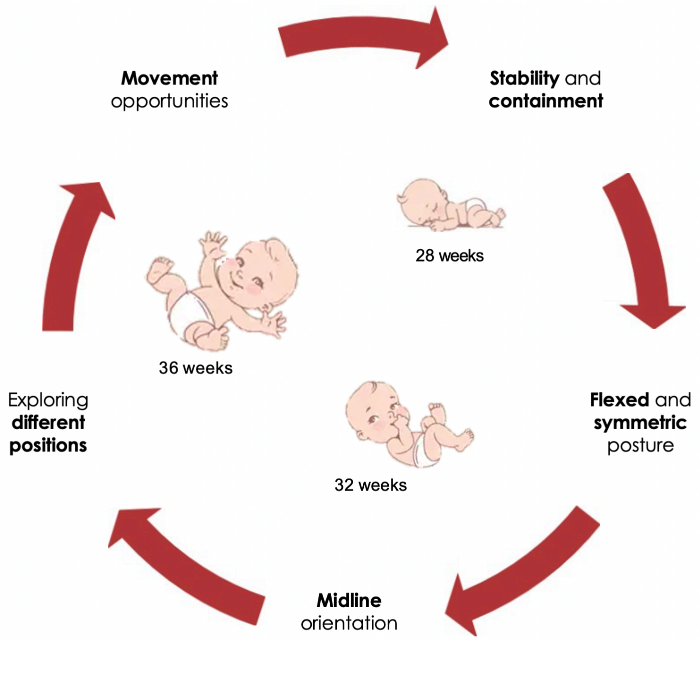

Positioning Strategies

SUPINE POSITION

- Allows **limbs movements.**
- Encourages **relational experiences with you.**
- May cause **greater instability and stress** as it's more difficult to bring legs and arms to the midline.

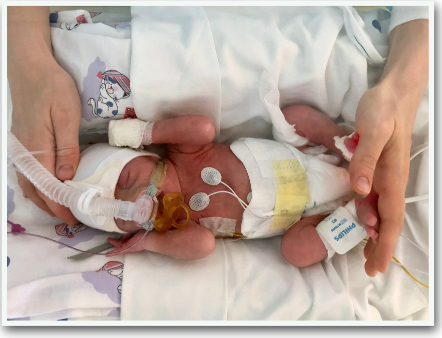

Promote a **semi-flexed and aligned position through containment**, provided with your hands, the nest or a blanket, safeguarding stability.

SIDELYING POSITION

- Promotes **flexion, symmetry** and **alignment.**
- Helps **midline orientation** (i.e. hand-to-hand).
- **Reduces behavioral instability.**
- May help with **bottle feeding.**

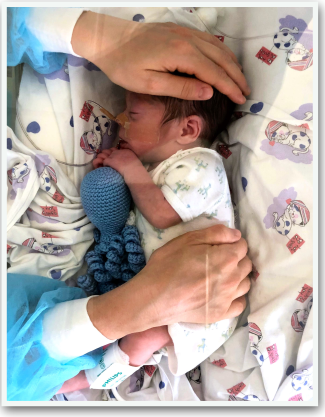

Provide **support for the trunk** with the nest and, if needed, with a front soft roll. Be careful to keep **head and trunk aligned.**

PRONE POSITION

- Improves **cardio-respiratory functioning.**
- **Reduces motor instability.**
- When the baby is older, it helps strengthen the muscles that will serve in the development of head control.

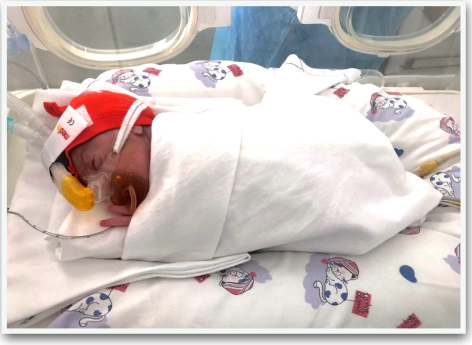

Take care to place a **support under the trunk.**

POSITIONING SUPPORTS FOR CONTAINMENT

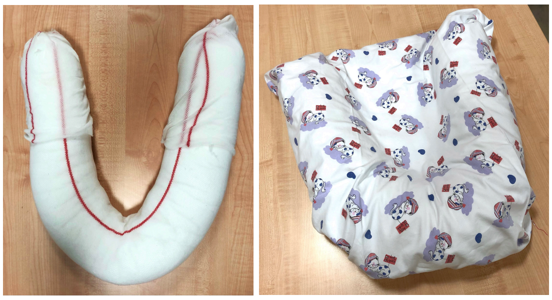

N E S T

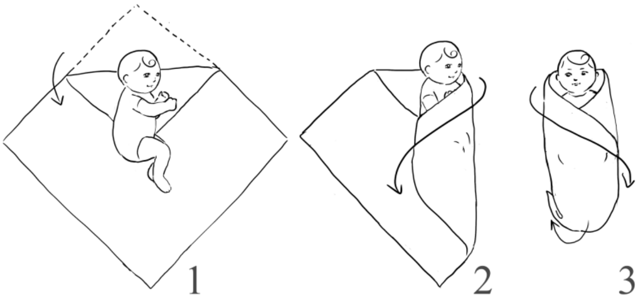

W R A P P I N G

Special thanks to babies and parents that helped us in this project!
