# Supplementary material for: Educational materials to empower parents of preterm infants within a family-centered early intervention in the NICU
Source: Front Pediatr. 2026 Jun 9;14:1823643. doi: 10.3389/fped.2026.1823643 (PMC13287061; doi:10.3389/fped.2026.1823643)
Supplement: Data Sheet 11 — Voice Listening - ENG. [file Datasheet11.pdf]

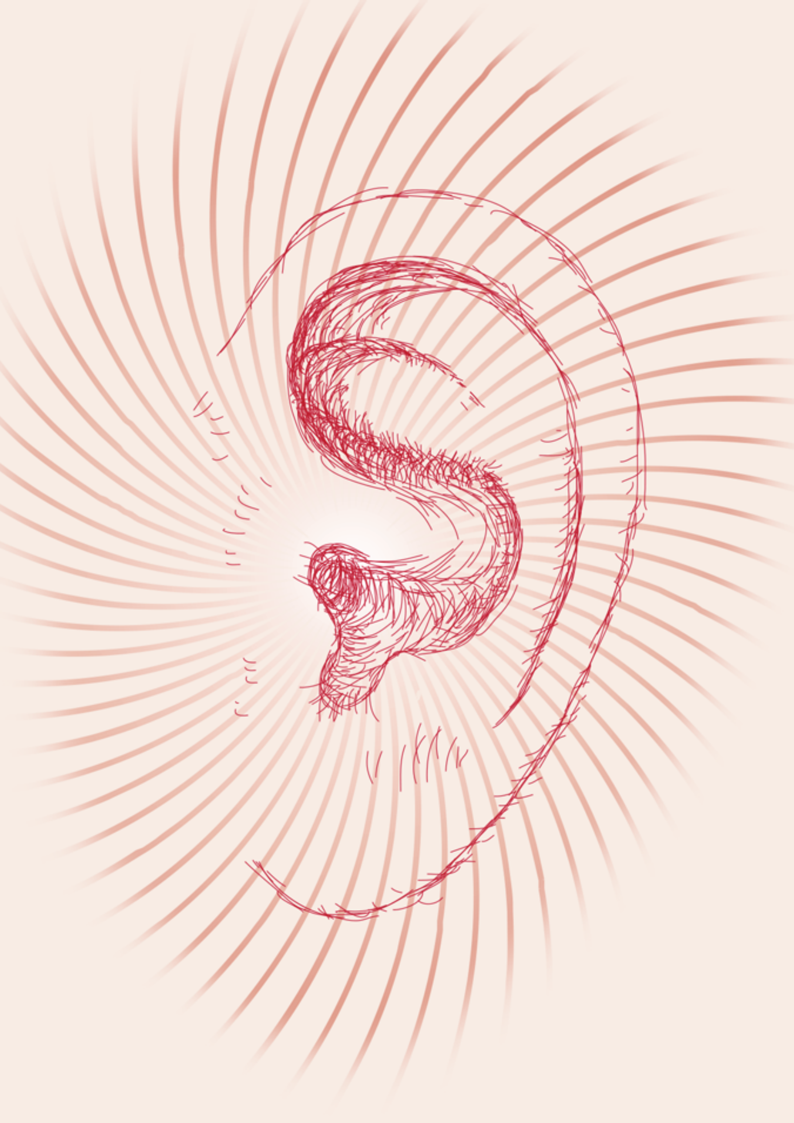

Bonfanti C, Fontana C, et al.

EARLY INTERVENTION

VOICE LISTENING

NICU, Fondazione IRCCS Ca' Granda
Ospedale Maggiore Policlinico, Milan, Italy

HOW

- Inside the incubator, **keeping your mouth close to the porthole**, or inside the crib; also during **kangaroo care** or while **holding** him/her in your arms.
- Try to keep the area near your baby **quiet**, **modulating**, as much as possible, the **sources of noise** near the infant.

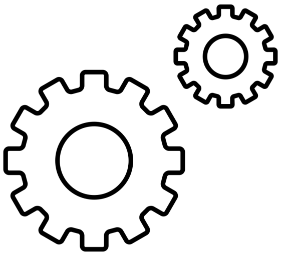

WHEN

- In a **quiet or alert behavioral state**, when showing comfort and stability signs, **to promote the transition from one behavioral state to another.**
- Your voice can also be used when your baby is **crying** or showing **stress signals,** to help him/her in self-regulation and stability.

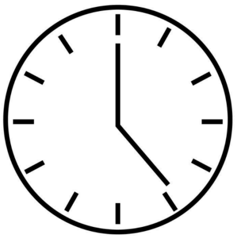

Ways to promote auditory and multisensory experiences through voice listening, supporting neurodevelopment

WHAT YOU CAN DO

Your baby's favorite sound is your voice!

- **Talk** gently to your baby
- **Read** a book or tell a story
- **Use your voice to create a melody,** using only few modulated sounds
- **Sing** him/her a lullaby or a song

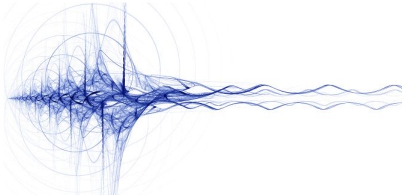

While promoting this experiences, try to **modulate your voice** keeping a **low volume**.

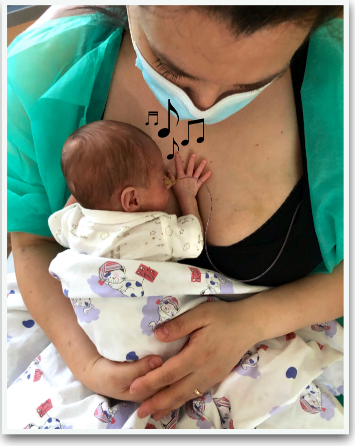

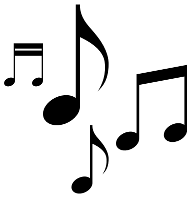

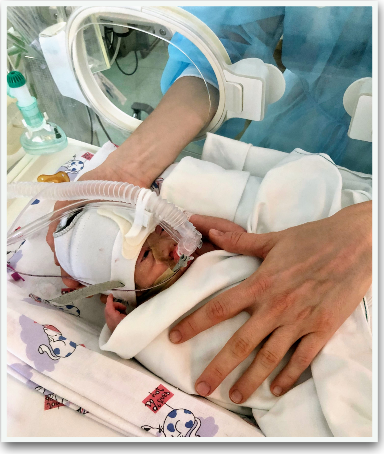

- *The use of your voice to talk to your baby can begin very early, as a way of **containment when it's not possibile for you to touch him/her.***
- *You can use your voice during other kind of experiences, such as **daily care** or kangaroo care.*

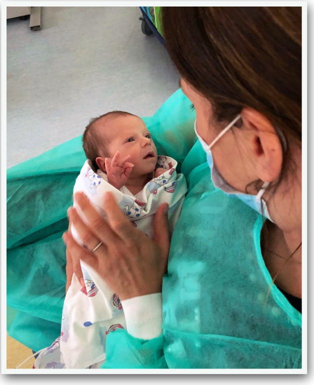

KEEP IN MIND

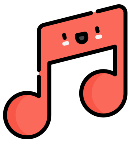

- *As you talk or read or sing to your baby, **watch for your baby's cues**.*
- ***Avoid too many different experiences.***
- *If the newborn shows stress signs:*
  - ✓ *try to **reduce the intensity** of the experience, and possibly **give a rest**;*
  - ✓ ***help him/her to handle sensory experiences** (i.e. offering a containment);*
  - ✓ *restart or suspend the activity, if necessary.*

Special thanks to babies and parents that helped us in this project!
