# Supplementary material for: Educational materials to empower parents of preterm infants within a family-centered early intervention in the NICU
Source: Front Pediatr. 2026 Jun 9;14:1823643. doi: 10.3389/fped.2026.1823643 (PMC13287061; doi:10.3389/fped.2026.1823643)
Supplement: Data Sheet 12 — Infant Massage - ENG. [file Datasheet12.pdf]

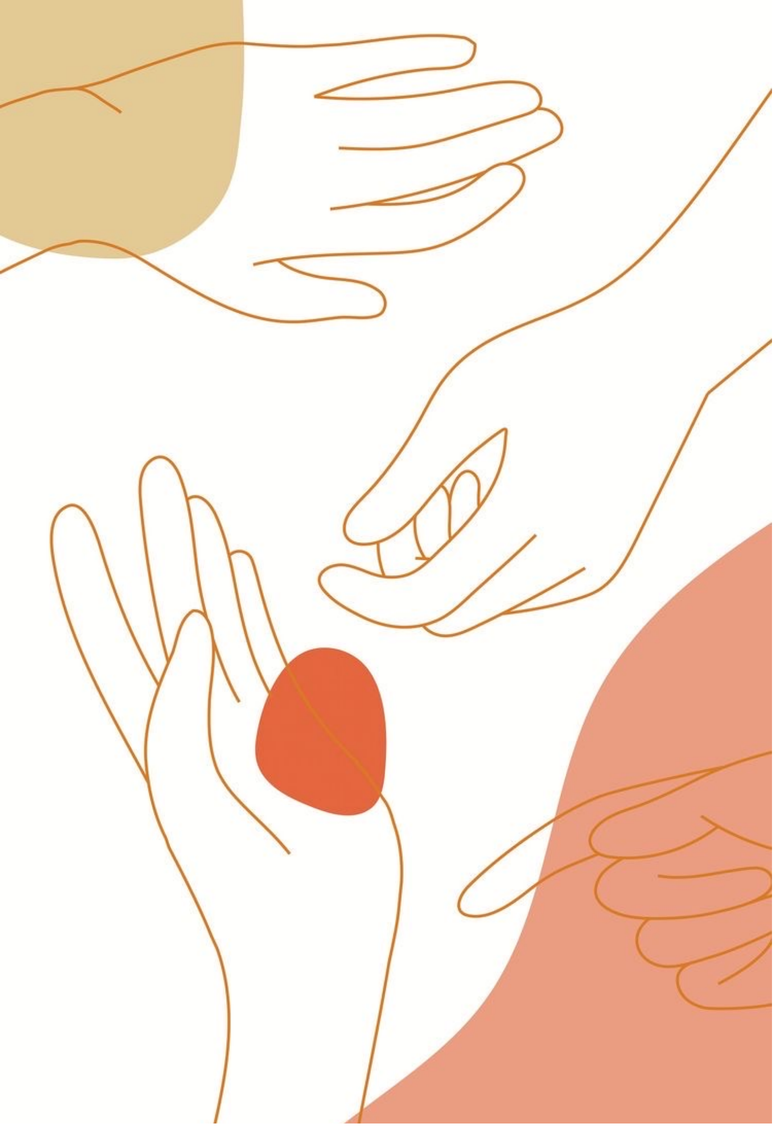

Bonfanti C, Fontana C, et al.

EARLY INTERVENTION

INFANT MASSAGE

NICU, Fondazione IRCCS Ca' Granda
Ospedale Maggiore Policlinico, Milan, Italy

HOW

- Prefer **prone position during Kangaroo Care.** Alternatively, **sidelying** in the incubator, in the crib, or in your arms, with support.

- **Skin-to-skin** if the baby tolerates it, or eventually with a blanket or the playsuit.

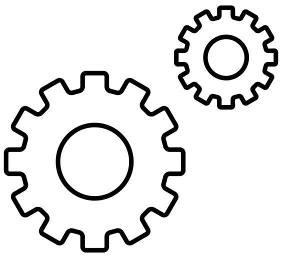

WHEN

- **Clinical stability**, once the infant is **30 weeks postmenstrual age.**

- **Quiet behavioral state**.

- **Not too close to the previous massage session** and, if possibile, **between feeding times**.

- For a **few minutes** during the day, when your baby **doesn't shows stress cues**.

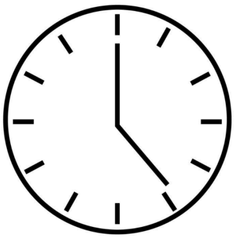

WAYS TO PROMOTE TACTILE AND MULTISENSORY EXPERIENCES THROUGH INFANT MASSAGE, SUPPORTING NEURODEVELOPMENT

INFANT MASSAGE

- Start applying gentle human touch, placing **one hand on your baby's head and the other on his/her back.**
- Keeping **one hand still**, begin to **move the other one slowly.**
- Continue with **both hands**: apply **light pressure** with the **palm and the fingers,** starting from the head and carrying on along the shoulders, back and legs.
- Try to maintain a **slow and steady pace**.
- **Gradually end** the proposal turning the massage into a firm and comforting touch.

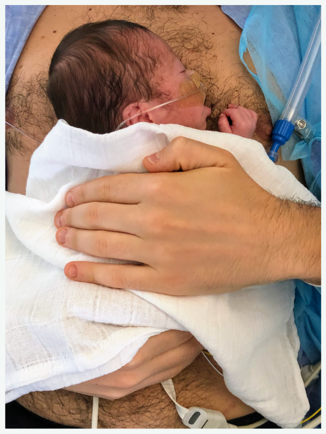

DURING KANGAROO CARE

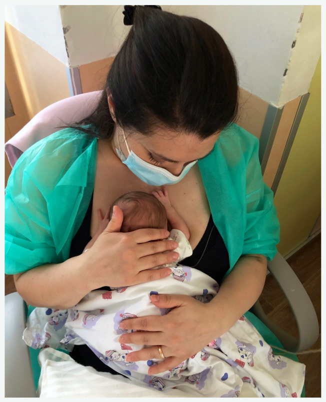

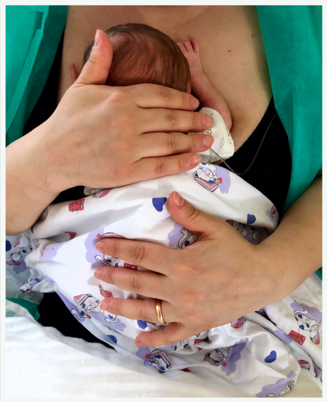

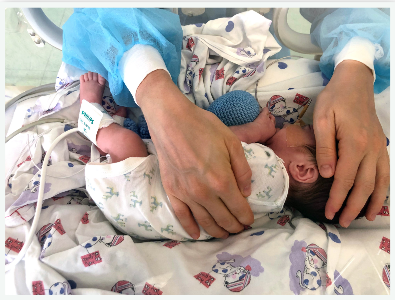

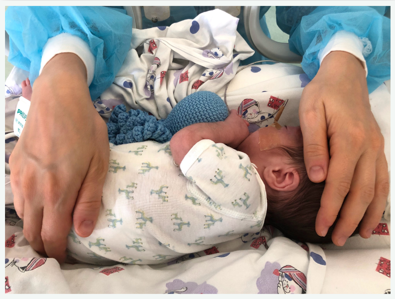

SIDELYING

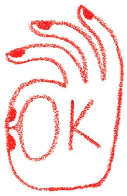

KEEP IN MIND

- Always remember to **read your baby's cues**: if he/she shows stress signals, use **a stable touch** to comfort him/her.
- Pay attention to **postural stability,** offering a positioning support with the nest and a towel.
- Try to begin and end the massage **as gradually as possible**.
- During massage, pay attention to behavioral signs. You can also **use your voice to talk or sing to your child.**

Special thanks to babies and parents that helped us in this project!
