# Supplementary material for: Educational materials to empower parents of preterm infants within a family-centered early intervention in the NICU
Source: Front Pediatr. 2026 Jun 9;14:1823643. doi: 10.3389/fped.2026.1823643 (PMC13287061; doi:10.3389/fped.2026.1823643)
Supplement: Data Sheet 13 — Visual Interaction - ENG. [file Datasheet13.pdf]

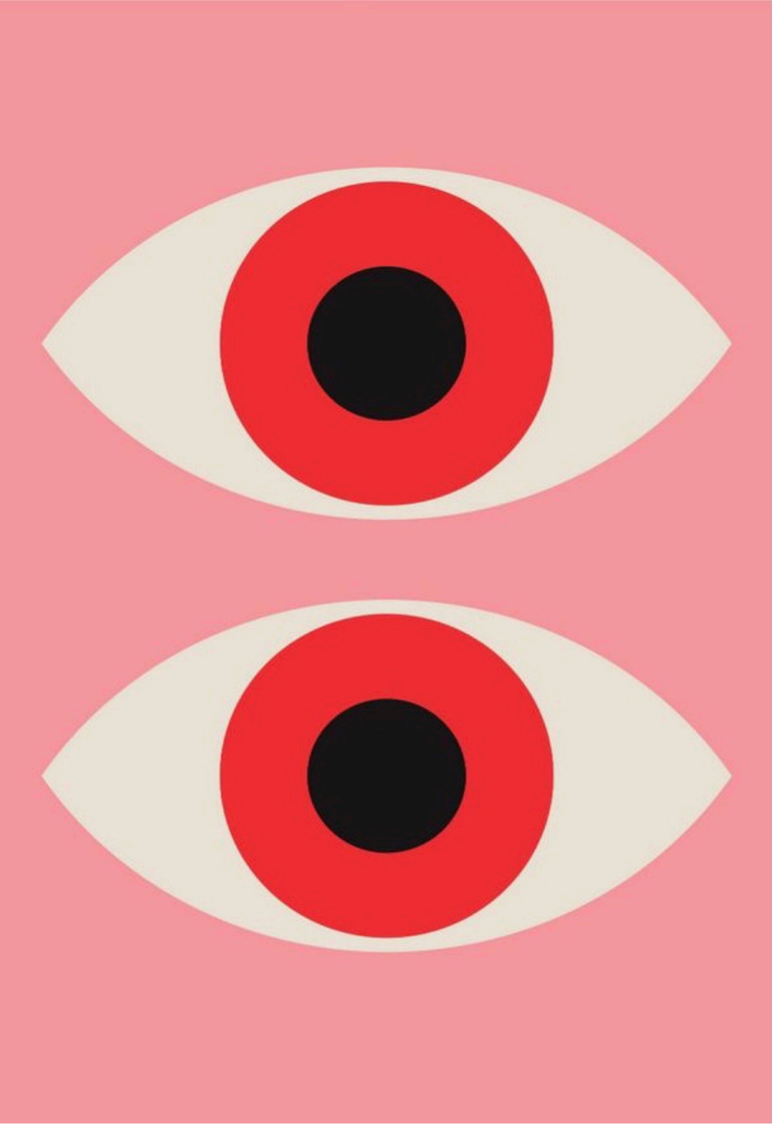

Bonfanti C, Fontana C, et al.

EARLY INTERVENTION

VISUAL INTERACTION

NICU, Fondazione IRCCS Ca' Granda
Ospedale Maggiore Policlinico, Milan, Italy

HOW

- **Supine position**, or alternatively sidelying, on your legs or in the crib.
- **Back light** source.
- Provide **containment** (i.e. wrapping) but be careful to leave the **head free to move on both sides.**

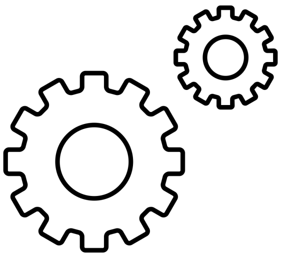

WHEN

- **Clinical stability**, starting from **34 weeks postmenstrual age.**
- **Alert** behavioral state.
- **For a few moments** during the day, when your baby shows availability signs.

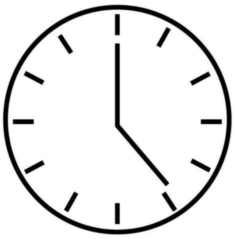

WAYS TO PROMOTE VISUAL AND MULTISENSORY EXPERIENCES THROUGH VISUAL INTERACTION, SUPPORTING NEURODEVELOPMENT

DIRECT FACE-TO-FACE INTERACTION

Move your face close to your baby's, remaining still and **allowing him/her to explore it.** If necessary, try to engage him/her using your **voice.**

Later, you can begin to **move slowly**, inviting the infant to follow you with his/her eyes.

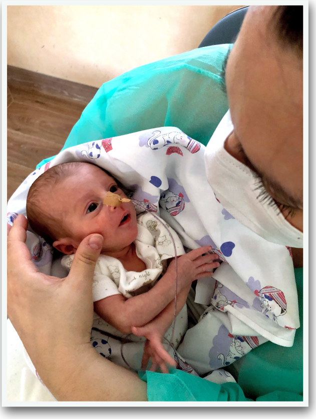

HIGH-CONTRAST TARGETS AND TOYS

Show to your child pictures, little books, soft toys with **high-contrast** (black and white, red and yellow) and **simple design** that can catch his/her attention.

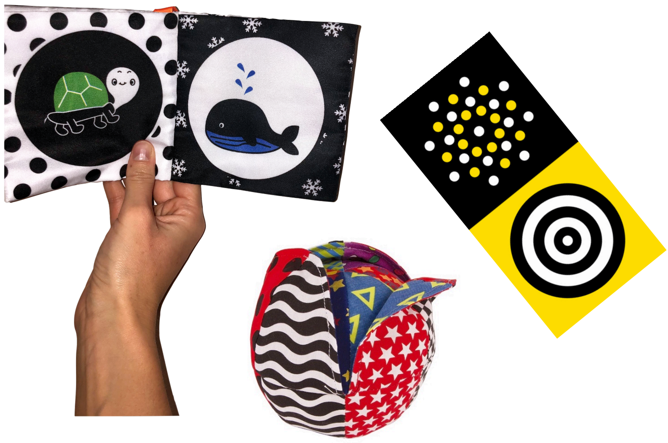

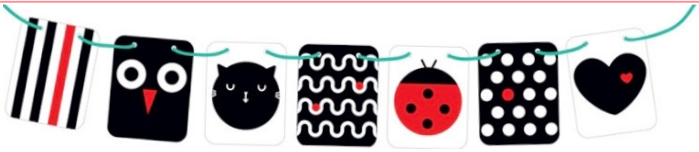

- Use **one thing at a time**, trying to keep a constant distance (**20-30 cm** from the face).
- Move **very slowly**, to help your baby following.
- Begin with **fixation** and only later with **horizontal tracking**. In the next weeks, he/she will be able to track vertically and in an arc.

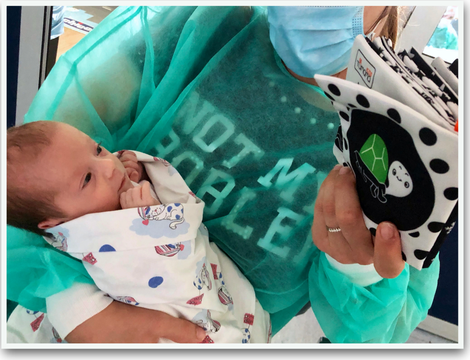

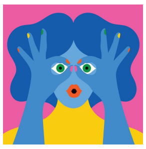

KEEP IN MIND

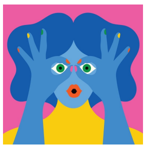

- Watch your baby's signals and try to **modulate the interaction**.
- Pay attention to be in a **quiet environment** with **low light** (coming from behind) and possibly **shield your baby's eyes** from direct lights with your hands or with a sheet.
- Try to safeguard **postural stability**, to promote quiet behavioral state, attention and interaction. If needed, you can help him/her by **increasing containment** or **gently holding the head.**

Special thanks to babies and parents that helped us in this project!
