# Supplementary material for: Educational materials to empower parents of preterm infants within a family-centered early intervention in the NICU
Source: Front Pediatr. 2026 Jun 9;14:1823643. doi: 10.3389/fped.2026.1823643 (PMC13287061; doi:10.3389/fped.2026.1823643)
Supplement: Data Sheet 14 — Finally...Back Home! - ENG. [file Datasheet14.pdf]

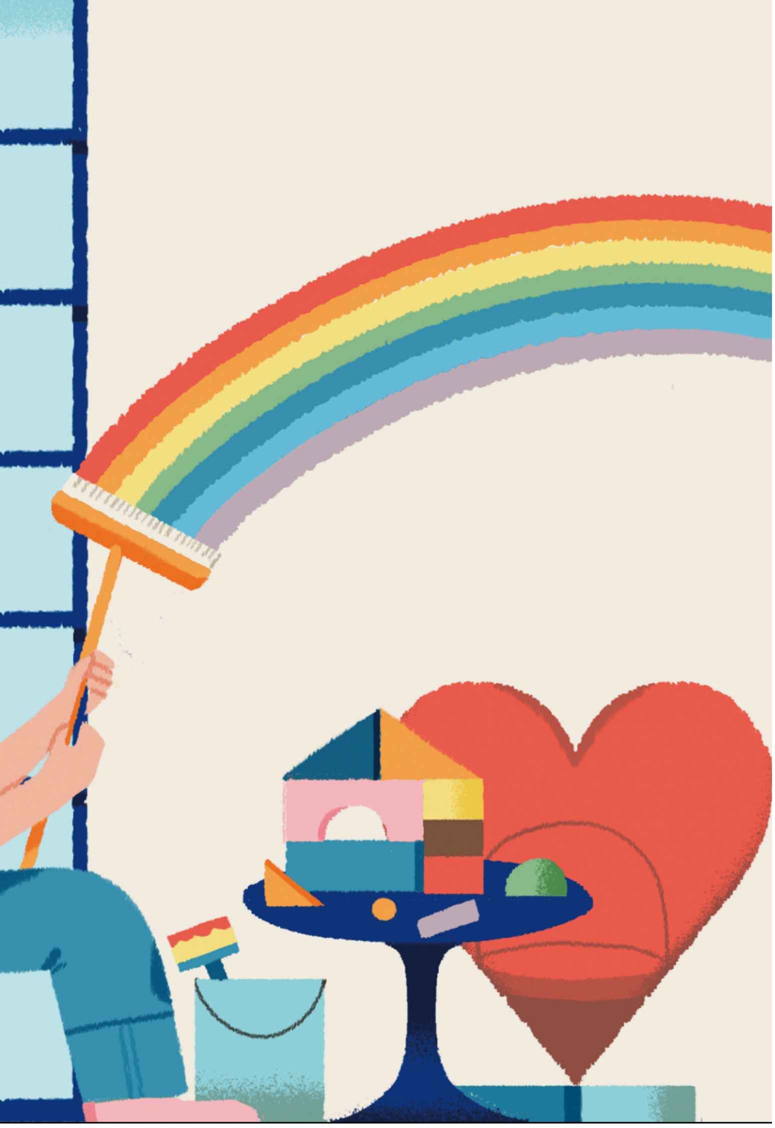

Bonfanti C, Fontana C, et al.

EARLY INTERVENTION

FINALLY… BACK HOME!

NICU, Fondazione IRCCS Ca’ Granda
Ospedale Maggiore Policlinico, Milan, Italy

WHAT TO KEEP IN MIND?

- Take care of your baby’s environment, trying to promote **appropriate sensory experiences**, avoiding excessive lights and sounds.

- Help your baby to organize his/her **sleep-wake cycle**, adjusting the activities throughout the day according to his/her needs.

- Encourage pleasant **play and interaction activities** during the day, promoting your baby’s attention and stability.

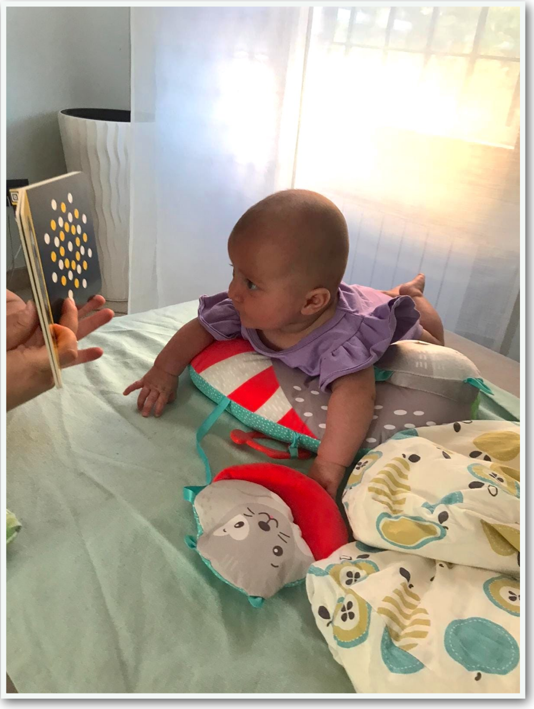

PROMOTING BABY'S WELL-BEING AND DEVELOPMENT AT HOME THROUGH INTERACTION AND PLAY

POSITIONING AND CONTAINMENT

- Provide **containment with a nest** and eventually a **light wrapping** to stabilize infant's posture.

- Offer **different postures** throughout the day: supine, prone and on the side. Change the position **even when holding the baby** in your arms.

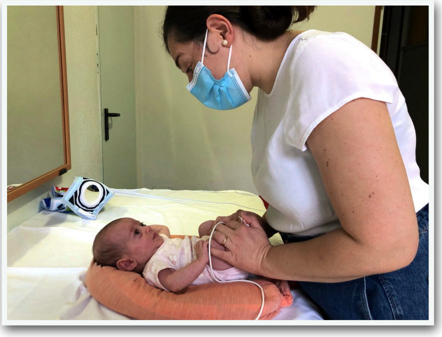

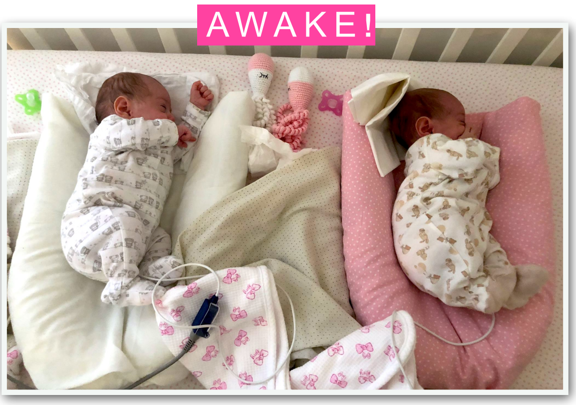

- Promote **prone and sidelying position for short periods**, only when your baby is in a **quiet or active behavioral state**, with support under the trunk and high-contrast toys.

- **Give movement opportunities** and **promote holding in your arms.**

WHICH ACTIVITIES CAN YOU DO TOGETHER?

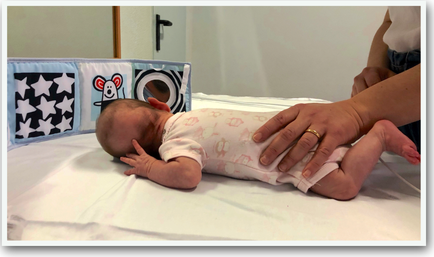

- **Visual interaction with your face,** for example you can sing, talk or read a book to your child.

- **Visual experiences** with pictures, little books, soft balls and toys with **high-contrast** (black and white, red and yellow).

- Foster **skin-to-skin contact** and **massage.**

WHEN?

- **Alert behavioral state.**

- **Calm and quiet** environment.

- **For a few moments** during the day, when your baby shows signs of availability.

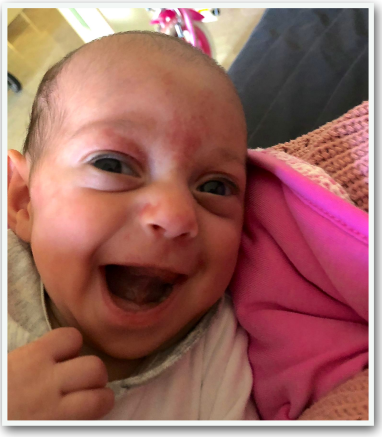

Special thanks to babies and parents that helped us in this project!
